# Computational molecular insights into ibrutinib as a potent inhibitor of HER2-L755S mutant in breast cancer: gene expression studies, virtual screening, docking, and molecular dynamics analysis

**DOI:** 10.3389/fmolb.2025.1510896

**Published:** 2025-03-19

**Authors:** Tamizhini Loganathan, C. George Priya Doss

**Affiliations:** Laboratory of Integrative Genomics, Department of Integrative Biology, School of BioSciences and Technology, Vellore Institute of Technology (VIT), Vellore, Tamil Nadu, India

**Keywords:** breast cancer, HER2-L755S mutant, lapatinib resistance

## Abstract

**Background:**

The proposed study integrates several advanced computational techniques to unravel the molecular mechanisms underlying breast cancer progression and drug resistance.

**Methods:**

We investigated HER2-L755S mutation through a multi-step approach, including gene expression analysis, molecular docking, and molecular dynamics simulations.

**Results and Discussion:**

By conducting a network-based analysis of gene expression data from breast cancer samples, key hub genes such as *MYC, EGFR, CDKN2A, ERBB2, CDK1, E2F1, TOP2A, MDM2, TGFB1,* and *FOXM1* were identified, all of which are critical in tumor growth and metastasis. The study mainly focuses on the ERBB2 gene, which encodes the HER2 protein, and its common mutation HER2-L755S, associated with breast cancer and resistance to the drug lapatinib. The HER2-L755S mutation contributes to both tumorigenesis and therapeutic failure. To address this, alternative therapeutic strategies were investigated using combinatorial computational approaches. The stability and flexibility of the HER2-L755S mutation were evaluated through comparative molecular dynamics simulations over 1000 ns using Gromacs in the unbound (Apo) state. Virtual screening with Schrodinger Glide identified ibrutinib as a promising alternative to lapatinib for targeting the HER2-L755S mutant. Detailed docking and molecular dynamics simulations in the bound (Holo) state demonstrated that the HER2-L755S-ibrutinib complex exhibited higher binding affinity and lower binding energy, indicating more stable interactions compared to other complexes. MM-PBSA analysis revealed that the HER2-L755S-ibrutinib complex had more negative binding energy than the HER2-L755S-afatinib, HER2-L755S-lapatinib, and HER2-L755S-neratinib complexes, suggesting that ibrutinib forms the most stable complex with favorable binding interactions.

**Conclusion:**

These results provide in-depth atomic-level insights into the binding mechanisms of these inhibitors, highlighting ibrutinib as a potentially effective inhibitor for the clinical treatment of breast cancer.

## Introduction

Breast cancer (BC) is the most prevalent cancer among women globally, though it can also affect men (Łukasiewicz et al., 2021). In 2020, over 2.3 million women were diagnosed with BC, making it the most common cancer, with an annual mortality rate of 30% (World Health Organisation, 2023; Arnold et al., 2022). According to GLOBOCAN statistics, BC accounted for 11.6% of all new cancer cases globally in 2022 (Bray et al., 2024). The American Cancer Society projects approximately 297,790 new cases of invasive breast cancer and 55,720 new cases of ductal carcinoma *in situ* in the US for 2024, with an estimated 43,170 deaths (American Cancer Society, 2023). In India, BC accounts for 25%–32% of female cancer cases, with 178,000 new cases reported annually as of 2020 (Mehrotra and Yadav, 2022). Approximately 50%–60% of BC cases in India are detected at a late stage, resulting in poorer outcomes and higher mortality rates. BC survival rates vary globally, influenced by the stage at diagnosis, access to healthcare, and socioeconomic factors. The 5-year global survival rate ranges from 85% to 90%, and the 10-year global survival rate exceeds 80% for early-stage BC but significantly decreases with advanced stages (Giaquinto et al., 2024). BC is classified into various types based on origin, hormone receptor status, and genetic characteristics. These include ductal carcinoma *in situ*, invasive ductal carcinoma, invasive lobular carcinoma, triple-negative breast cancer (TNBC), hormone-receptor-positive BC, and inflammatory BC. Each subtype has distinct treatment approaches and prognoses (Mayo Clinic, 2018; Zubair et al., 2021). BC risk factors include genetic, environmental, and lifestyle factors such as gender, age, family history, genetic mutations, hormonal factors, and diet (Feng et al., 2018).

BC is often driven by genetic mutations that affect cell growth and division, with some mutations being inherited and others occurring spontaneously (Breast cancer: MedlinePlus Genetics, 2021). Recently, gene expression analysis has become crucial in identifying molecular drivers in diseases like BC (Alam et al., 2022; Alberts et al., 2015). Researchers can identify critical targets and pathways linked to disease progression and treatment response by examining gene expression differences between patient samples. Investigating mutations within these targets provides deeper insights into specific alterations that may drive resistance mechanisms (Waarts et al., 2022). This approach enhances our understanding of the disease’s molecular landscape and the development of targeted therapies to overcome resistance, ultimately leading to more effective and personalized treatment strategies.

The *ERBB2* (*HER2*) gene, part of the ERBB family of receptor tyrosine kinases, plays a crucial role in cell growth, differentiation, and survival (Tan and Yu, 2013). Mutations and amplifications in *HER2* are commonly associated with several cancers, including BC, leading to HER2 protein overexpression and uncontrolled cell proliferation (Lee et al., 2019; Michael et al., 2023). The HER2 protein has four functional domains: extracellular, transmembrane, juxtamembrane, and kinase domains (Pahuja et al., 2018). The kinase domain is particularly critical, as it phosphorylates tyrosine residues on the receptor and downstream signaling proteins, activating key pathways like PI3K/AKT/mTOR and RAS/RAF/MEK/ERK (Jiao et al., 2018; Sudhesh Dev et al., 2021). The mutation frequency of the HER2 protein was analyzed using the COSMIC database, revealing it ranks 14th in the context of BC (Tate et al., 2018). Further analysis identified various mutations across different domains of the HER2 protein. Mutations in the kinase domain of HER2 are the most frequent and often oncogenic. Notable kinase domain mutations, such as L755S, V777L, D769Y, D769H, I767M, V842I, and T798M, lead to constitutive activation of the receptor, driving cancer progression (Subramanian et al., 2019). Among these, the HER2-L755S mutation is particularly deleterious and supported by other resources (ERBB2 - My Cancer Genome, 2024; Uchida et al., 2021) (Supplementary Figure S1). This mutation continues to drive cancer progression and is a target for HER2 inhibitors like neratinib and afatinib (Swain et al., 2022). Additionally, L755S and T798M mutations confer resistance to some HER2-targeted therapies, such as trastuzumab and lapatinib (Li et al., 2019). Drug resistance in BC, particularly with HER2 mutation (L755S), occurs through various biological mechanisms that allow cancer cells to evade targeted therapies. Resistance can be primary (innate) or secondary (acquired), where resistance develops after an initial response to treatment (Sharma et al., 2017). Understanding these mechanisms and exploring alternative strategies are crucial for improving patient outcomes.

The integration of combinatorial bioinformatics approaches, including gene expression analysis, virtual screening, docking, and dynamics analysis, has revolutionized the drug discovery process (Ismail et al., 2024; Suvarna et al., 2024; Helal et al., 2022). This study aims to thoroughly investigate the impact of the HER2-L755S mutation and its drug-binding efficiency on four drugs: lapatinib (drug-resistant) and afatinib, neratinib, and ibrutinib (drug-sensitive) for BC. The initial bioinformatics analysis combined with gene expression identifies key hub genes and maps predominant mutations with external databases. Identifying these genes and mutations is crucial for further cancer research. Subsequently, virtual screening and molecular dynamics were employed to explore possible binding interactions and ligand stability, identifying promising drug candidates for HER2-L755S. This study is the first comprehensive investigation demonstrating the compound’s ability to interact with HER2, laying the groundwork for the potential development of an effective bioinformatics pipeline in the drug discovery process towards BC.

## Methods

### Collection of gene expression datasets

**FIGURE 1:**
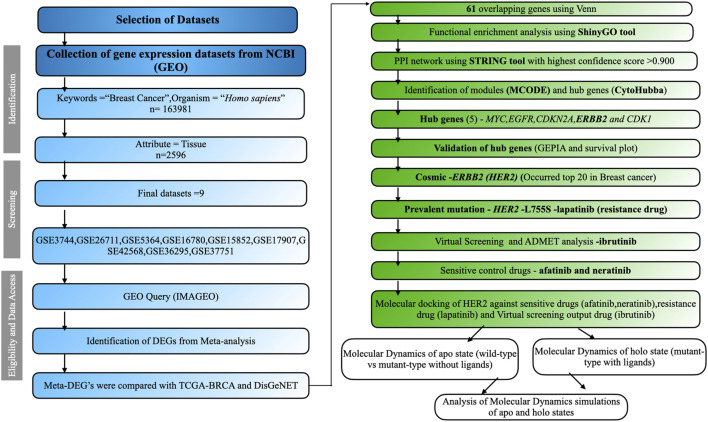
The comprehensive workflow of this study. Datasets were screened based on inclusion and exclusion criteria using the PRISMA workflow. The overall analysis includes identifying DEGs, performing functional enrichment analysis, constructing the PPI network, screening modules, identifying hub genes, and conducting structural studies.

We employed the keywords “Breast Cancer” to explore the GEO, a public platform, and retrieve gene expression datasets (Clough and Barrett, 2016). GEO databases allow for the selection of datasets according to predefined criteria by applying strict filtering standards and developing a method to identify relevant information. The research focused on the organism category (*Homo sapiens*) and the gene expression analysis using microarrays. The chosen traits involved tissue samples from cancerous tissues as well as normal neighboring tissues. We selected datasets containing normal adjacent samples as well as diseased samples, ensuring there were a minimum of five samples from patients in cancer stages I, II, and III, utilizing the affymetrix platform (Irizarry et al., 2003). Criteria for exclusion encompassed research on cell lines, datasets involving treatment interventions, and studies lacking normal healthy or tumor samples. The analysis of data involved pinpointing differentially expressed genes (DEGs) from various datasets, building a protein interaction network, evaluating DEG functions, utilizing CytoHubba to find hub genes, recognizing mutations, and performing structural investigations. The complete process is illustrated in Figure 1.

### Identification of DEGs

ImaGEO was employed to detect DEGs in BC using pre-normalized microarray data (Toro-Domínguez et al., 2018). By combining effect sizes from all datasets, DEGs were integrated using a random effect model. Genes were considered significant if they had a p-value < 0.01, corrected using the false discovery rate (FDR). DEGs with a z-score > 3 were classified as upregulated, while those with a z-score < −3 were classified as downregulated. Venny was an online tool used to identify and visualize overlapping genes from TCGA-BRCA, DisGeNET (Piñero et al., 2016), and Meta-DEG from BC. A subsequent functional enrichment analysis was conducted using these shared overlapped genes.

### Enrichment analysis

In order to identify the underlying Cellular Components (CC), Biological Processes (BP), Molecular Functions (MF), Transcription Factors (TF), and crucial signaling pathways, the DEGs were examined using ShinyGO (Ge et al., 2019) for Gene Ontology (GO) and pathway enrichment. Biological terms with a p-value less than 0.01, a minimum count of 3, and an enrichment factor over 1.5 were sorted.A p-value of less than 0.05 was used as a threshold criterion to select significant enriched GO keywords and pathways.

### Building PPI networks and identifying hub genes

The STRING web tool was used to retrieve gene interaction data and build a PPI network (Szklarczyk et al., 2022). The selection of genes with an interaction score greater than 0.9 indicated high confidence. The MCODE plugin was used to identify modules or clusters displaying strong co-expression patterns with other genes and relevant biological functions (Bader and Hogue, 2003). The gene information acquired in network form was then screened using Cytoscape software and the CytoHubba plugin (Chin et al., 2014). PPI connections were managed using the MCC technique in Cytoscape software. The top ten nodes with the most interactions were identified and classified as hub genes.

### Validation on hub genes

Hub genes were validated across multiple cancer types using the GEPIA webserver (Tang et al., 2017), which was utilized to analyze gene expression and retrieve data for specific genes. GEPIA is widely recognized for its capability to compare gene expression levels between normal and tumor samples. Kaplan-Meier plots were generated for survival analysis, assessing differential gene expression between tumor and normal tissues.

Gene expression data and overall survival information from TCGA were analyzed using the KM-plotter, an online survival analysis tool that stratifies patient samples into two groups. The hub gene signature score was calculated based on the average log2 (TPM + 1) value. The expression threshold for both cancer tissue types was set at 0.01 (p-value), while |Log2FC| was fixed at 1.

This tool enables users to select their cancer type for overall survival analysis. Statistical significance was evaluated using the log-rank test, also known as the Mantel-Cox test. Additionally, the Cox proportional hazard ratio and the 95% confidence interval can be displayed in survival plots. Kaplan-Meier graphs (Lánczky and Győrffy, 2021) illustrate the survival status of key genes along with the computed log-rank p-value across various cancer types.

### Gene prioritization and mutation selection using COSMIC database

Gene prioritization and mutation-selection were performed using the COSMIC database (Tate et al., 2018). Filters applied included tissue type (e.g., breast cancer), mutation type (e.g., SNP), and sample source (e.g., primary tumor). Mutations were reviewed and ranked by prevalence and biological impact. The HER2 L755S mutation, frequently identified in BC and associated with drug resistance, was selected for further analysis.

### Structure modeling of HER2 protein

The three-dimensional (3D) structure of the HER2 protein was acquired from the Protein Data Bank (PDB) (Burley et al., 2018), using the wild-type (WT) structure with PDB ID: 3PP0 for subsequent analysis (Aertgeerts et al., 2011). The missing residues in the crystal structure were modeled using SWISS-MODEL (Waterhouse et al., 2018). After modeling, SWISS-MODEL was used for energy minimization to ensure the stability and compatibility of the newly added residues with the existing structure. The overall structure, including the newly modeled residues, was assessed using metrics like QMEAN to estimate accuracy and reliability (Waterhouse et al., 2018). The modelled structure was validated using Ramachandran plot (Supplementary Figure S2). The finalized modeled structure was downloaded in.pdb format. This structure has two chains; chain A was selected for further analysis. Furthermore, the mutation (L to S) at position 755 in HER2 was introduced using the Swiss PDB Viewer (Kaplan and Littlejohn, 2001). The prepared models, Apo WT and MT L755S, were considered for further analysis. The PyMOL tool were used to visualize the protein structure (Schrödinger and DeLano, 2020).

### Virtual screening and ADMET analysis

The tyrosine kinase library was sourced from Selleck Chemicals (https://www.selleckchem.com). The protein structure (HER2-L755S) was prepared by adding hydrogen atoms, refining loop regions, optimizing hydrogen bond assignments, and minimizing energy using the OPLS-2005 force field with the Protein Preparation Wizard (Schrodinger) (Roos et al., 2019). The Glide-grid was generated using the Receptor Grid Generation module. LigPrep processed the drug library to generate multiple conformers and ionization states at pH 2.0, utilizing the OPLS-2005 force field. Epik v5.3 generated ligand molecules at pH 7.0 ± 2, excluding high-energy ionization/tautomer states for reliability (Shelley et al., 2007). Structure-based virtual screening was performed after compounds were evaluated using Qikprop v6.5 and Lipinski’s criteria (Lipinski, 2004). Docking was conducted using Glide, employing three protocols: extra precision (XP), standard precision (SP), and high-throughput virtual screening (HTVS). XP was utilized to bind ligands to the receptor, generate three poses, and provide optimal scoring results (Friesner et al., 2006). MM-GBSA predicted the binding free energy of protein-ligand complexes, enhancing initial docking accuracy (Genheden and Ryde, 2015). This approach improves screening reliability, reducing false positives. Understanding pharmacokinetics is vital for effective drug development. ADMET properties (absorption, distribution, metabolism, excretion, and toxicity) were computationally assessed using the SwissADME web server to evaluate pharmacological and carcinogenic properties (Daina et al., 2017).

### Molecular docking analysis

The mutated modeled structure (HER2-L755S) was selected as the target protein for further analysis. Drug compounds with IDs (afatinib: 10184653, lapatinib: 208908, neratinib: 9915743, and ibrutinib: 24821094) were sourced from the PubChem database (Kim et al., 2015). Molecular docking analysis was performed using Autodock4.2.6 software to validate the protein-drug interactions (Forli et al., 2016). The dimensions of the grid box were set to 60 Å × 60 Å × 60 Å, and the center of X (8.559 Å), Y (17.570 Å), and Z (22.958 Å). The process involved protein preparation, ligand preparation, grid generation, and docking analysis. Active site residues, identified from the protein’s 3D structure, were chosen for the study (Aertgeerts et al., 2011). Autogrid4 and autodock4 software runs were used to prepare the receptor protein for docking and collect the findings. Based on hydrogen-bond interactions, the optimal complex was chosen from each drug’s triple docking. The binding energies of the drug compounds to the target proteins were determined using Autodock; more significant negative binding energy scores denoted more favorable interactions. Protein-drug interactions were visualized using the PLIP tool (Salentin et al., 2015).

### Molecular dynamics simulations

A 1000 ns comparative molecular dynamics simulation (MDS) was executed using the CHARMM force field. This simulation encompassed both the Apo (WT and MT HER2-L755S) and the Holo state with four complexes: HER2-L755S-afatinib, HER2-L755S-ibrutinib, HER2-L755S-lapatinib, and HER2-L755S-neratinib, utilizing Gromacs software (Abraham et al., 2015). SwissParam was used to create topological files for the ligand (Zoete et al., 2011). Three chloride ions were introduced to the system to bring it into equilibrium while keeping the temperature (300 K), pressure, and particle count unchanged. For solvation, the TIP3P water model was used, and the Berendsen thermostat was used to regulate the temperature (Berendsen et al., 1984). Every atom was placed at least 1 nm apart from the box’s edges.Energy minimization was conducted using the steepest descent method, with the temperature gradually increased from 0 K to 300 K during heating. The MDS consisted of three stages: heating, equilibration, and production. Energy minimization and equilibration steps were performed in both NVT and NPT ensembles (nsteps = 50,000), followed by a 1,000 ns MDS run. Covalent bond constraints were applied using the Linear Constraint Solver (LINCS) method (Hess et al., 1997), and electrostatic interactions were calculated using the Particle Mesh Ewald (PME) method (Essmann et al., 1995), with default cutoff radii for other interactions. The trajectory of each MD simulation was examined utilizing GROMACS tools (Van Der Spoel et al., 2005). Various structural features, including Root Mean Square Deviation (RMSD), Root Mean Square Fluctuation (RMSF), Radius of Gyration (Rg), intramolecular hydrogen bonds, and Solvent Accessible Surface Area (SASA), were assessed using gmx tools. Hydrogen bond occupancy analysis was conducted using Visual Molecular Dynamics (VMD) (Humphrey et al., 1996). VMD identified and calculated the occupancy of hydrogen bonds over the MDS trajectory, providing insights into the stability and interaction patterns within the complexes. The analysis involved specific distance (3.0 Å) and angle criteria (angle cutoff −20^◦^) for hydrogen bond detection, and occupancy was quantified to reveal the persistence of these bonds across the trajectory frames. This information characterized the hydrogen-bonding network, essential for understanding binding stability and structural changes in the complexes.

### Molecular mechanics Poisson–Boltzmann surface area

Molecular Mechanics Poisson–Boltzmann Surface Area (MM-PBSA) calculations are instrumental in integrating protein-ligand binding free energies within high-throughput MDS. These calculations encompass van der Waals, electrostatic, polar solvation, solvent accessible surface area (SASA), and binding energies. In this study, we employed the g_mmpbsa tool developed by Kumari et al. (2014). The MM-PBSA calculation was executed in a single step using the parameter file and the protein-ligand MD trajectory data. The van der Waals method was utilized via the MmPbsaStat.py Python script within the g_mmpbsa package to compute various energies, including binding, polar solvation, electrostatic, and SASA. Energy decomposition for the five complexes was performed using the MmPbSaDecomp.py script, and the results were visualized with the XmGRACE program.

### Principal component analysis (PCA)

Essential dynamics involves examining the fundamental motions of a biomolecule or group of molecules crucial for their biological activity. PCA is commonly employed to identify significant collective motions, extract essential dynamics from MD trajectories, and understand their relevance (Amadei et al., 1993). The elements of atomic vibration that contribute to this coordinated motion were shown by the time-averaged projection. The Gibbs free energy landscape was established using the gmx sham tool.

## Results

### Identification of DEGs in meta-analysis

**FIGURE 2:**
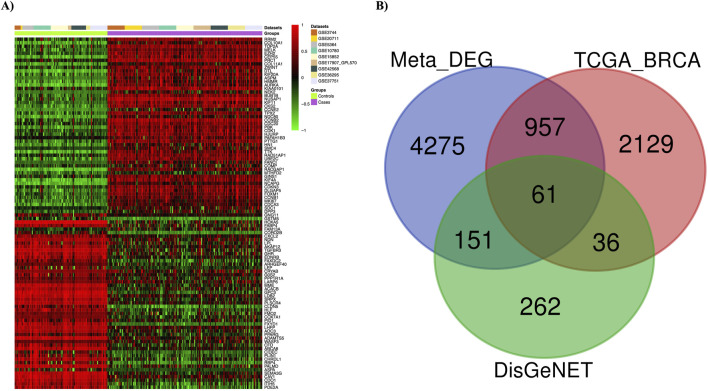
Meta-analysis results. **(A)** Differentially expressed genes (DEGs) in breast cancer are depicted using a heatmap. **(B)** The Venn diagram shows 61 overlapping DEGs among three different sets (Meta-DEGs, TCGA-BRCA, and DisGeNET(BC)).

Nine gene expression datasets were collected from the GEO database under stringent criteria. The datasets—GSE3744, GSE26711, GSE5364, GSE16780, GSE15852, GSE17907, GSE42568, GSE36295, and GSE37751—were processed and analyzed using the ImaGEO tool, with detailed information in Supplementary Table 1. Data quality was visualized via boxplots (Supplementary Figures S3, S4). DEGs were integrated using a random effect model, aggregating effect sizes from all datasets. Significant genes were identified with an FDR-p ≤ 0.05, corrected by the FDR method. Genes with a z-score > 3 were considered upregulated, while those with a z-score < −3 were considered downregulated. The meta-analysis identified 5,444 DEGs, visualized with a statistical heatmap in Figure 2A. DEGs were compared to the TCGA-BRCA and DisGeNET databases, identifying 61 overlapping genes. These common DEGs, illustrated in the Venn diagram in Figure 2B, were used for functional analysis.

### Functional enrichment analysis

**FIGURE 3:**
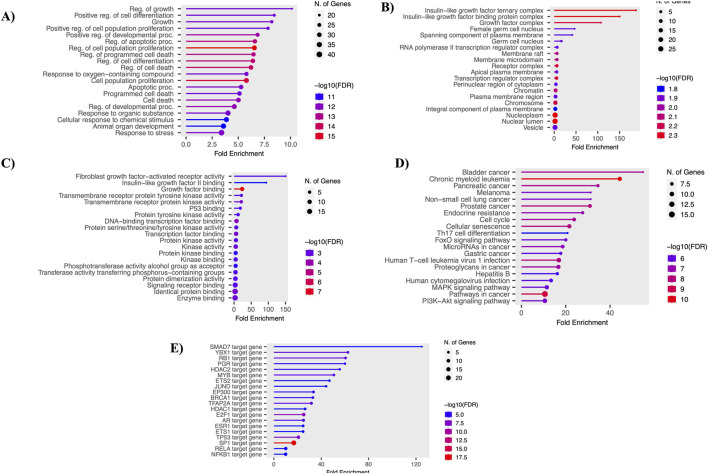
Functional enrichment analysis. **(A)** Gene ontology - biological processes. **(B)** Gene ontology - molecular function. **(C)** Gene ontology - cellular components. **(D)** Gene ontology - KEGG pathways. **(E)** Gene ontology - transcription factors.

Using the shinyGO tool, the functional enrichment of the 61 overlapping genes was analyzed. The analysis focused on various essential functions, including GO-BP, GO-MF, GO-CC, KEGG, and TF. Each analysis was visualized using lollipop plots with a p-value ≤ 0.05. Key GO-BP functions identified were “Regulation of growth,” “Positive regulation of cell differentiation,” and “Regulation of apoptotic process.” In GO-CC, functions such as “Growth factor complex” and “Insulin-like growth factor ternary complex” were noted. GO-MF functions included “Protein tyrosine kinase activity,” “Growth factor binding,” and “Transcription factor binding.” Crucial pathways identified included “Endocrine resistance,” “Cell cycle,” “FoxO signaling pathway,” “MAPK pathway,” and “PI3K-Akt signaling pathway.” Top enriched transcription factors (TFs), such as *E2F1*, *MYB*, and *TP53*, play vital roles in controlling gene expression and impacting various biological processes and development. These results were visualized using bar plots, as shown in Figure 3.

### PPI network and hub gene analysis

**FIGURE 4:**
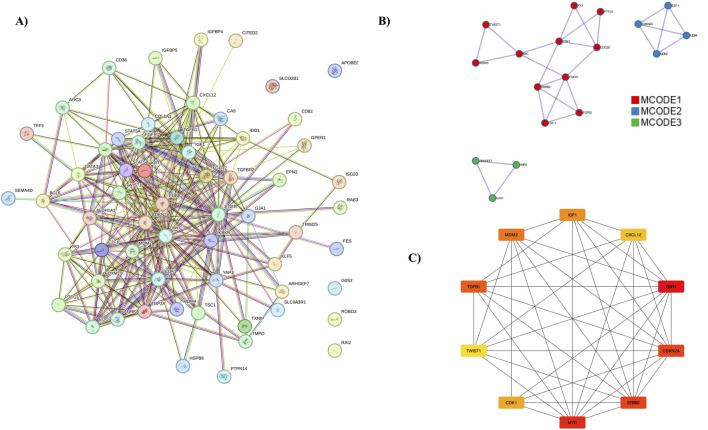
PPI Network Analysis and Hub Genes. **(A)** The protein-protein interaction network, represented using the STRING database. **(B)** MCODE analysis identifying three clusters. **(C)** Representation of hub genes.

The PPI network was constructed using the STRING database, focusing on interactions with a confidence score above 0.9. This network was then imported into the Cytoscape tool, with the entire network visualized in Figure 4A. Three distinct clusters were identified within the network, as shown in Figure 4B. Hub genes were identified using the MCC algorithm, with results displayed in Figure 4C. The identified hub genes—*MYC*, *EGFR*, *CDKN2A*, *ERBB2*, *CDK1*, *E2F1*, *TOP2A*, *MDM2*, *TGFB1*, and *FOXM1*—play crucial roles in breast cancer (BC) progression and development. Further validation of these hub genes was performed using the GEPIA server tool. The expression of the *ERBB2* gene was found to be higher in BC compared to other cancers, with the expression patterns illustrated. Survival analysis using KM plots was conducted for the *ERBB2* gene in both cancers. These findings are detailed in Supplementary Figure S5.

### Virtual screening and ADMET analysis

Virtual screening analysis was conducted using Glide Schrodinger software, focusing on the mutant HER2-L755S as the primary structure. The tyrosine kinase drug library (654 compounds) was utilized to identify potential inhibitors for the HER2-L755S protein. Initially, 344 compounds were filtered using the Qikprop and Lipinski modules. The final compound poses were selected after applying the extra precision (XP) algorithm for accurate screening. Ensuring the efficacy and reliability of the identified compounds involved assessing pharmacokinetics and toxicity characteristics. Parameters such as p-glycoprotein inhibition, hepatotoxicity, carcinogenicity, and blood-brain barrier absorption were examined. CNS permeability, indicated by CNS > −2, suggested central nervous system penetration. None of the compounds displayed carcinogenic or toxic profiles in AMES toxicity or carcinogenicity studies. The chosen compounds exhibited favorable responses according to the Lipinski rule of five, which considers hydrogen bond donors, acceptors, and ligand molecule surface area. A comprehensive summary of the ADMET analysis and virtual screening of ibrutinib outcomes is provided in Supplementary Table 2.

### Molecular docking results

**FIGURE 5:**
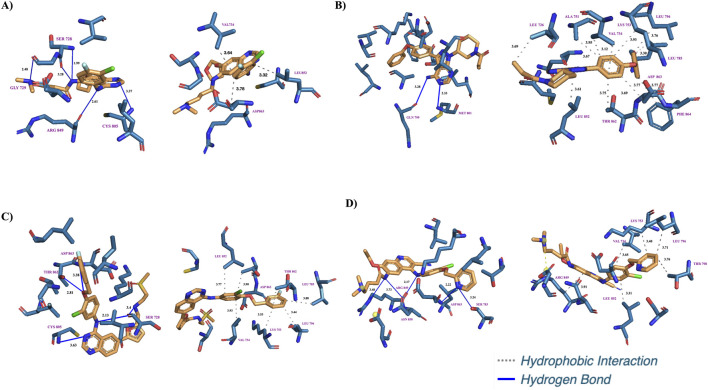
Molecular Docking Analysis. The molecular docking analysis of four different complexes of the mutated HER2 L755S structure. **(A)** HER2-L755S-afatinib. **(B)** HER2-L755S-ibrutinib. **(C)** HER2-L755S-lapatinib. **(D)** HER2-L755S-neratinib. Results were generated using the PLIP tool.

Molecular docking analysis was conducted using the conventional ligands lapatinib (drug-resistant), afatinib, and neratinib (drug-sensitive), and ibrutinib from virtual screening. Detailed docking results, including hydrogen bond and hydrophobic interactions, are provided in Table 1. Lower binding energy between protein-ligand complexes indicates a more favorable and stable interaction. The HER2-L755S-ibrutinib complex showed the lowest binding energy of −10.4 kcal/mol, followed by HER2-L755S-lapatinib at −9.7 kcal/mol. Other binding energies for the Holo complexes were HER2-L755S-afatinib at −7.7 kcal/mol and HER2-L755S-neratinib at −9.0 kcal/mol. The standard drug-sensitive complexes (HER2-L755S-afatinib and HER2-L755S-neratinib) displayed lower binding energies than the HER2-L755S-ibrutinib complex. Detailed molecular docking information is provided in Supplementary Table 3. The 2D interactions of the ligand-complex structures were visualized using the PLIP tool (Figure 5).

**TABLE 1:** Molecular docking analysis of HER2-L755S-drug complexes.

Protein_name	Ligand_name	Docking score (kcal/mol)	Number of h-bonds	Hydrogen Bonds residues and distances (Å)	Hydrophobic Interaction residues and distances (Å)
HER2	Afatinib	−7.7	5	SER 728A-3.28 SER 728A-1.99 GLY 729A-2.48 CYS 805A-3.37 ARG 849A-2.61	VAL 734A-3.64 LEU 852A-3.32 ASP 862A-3.78
HER2	Ibrutinib	−10.4	2	GLN 799A-3.28 MET 801A-2.33	LEU 726A- 3.69 VAL 734A-3.95 VAL 734A-3.12 ALA 751A-3.67 LYS 753A-3.93 LEU 785A-3.30 LEU 796A-3.76 LEU 852A-3.61 THR 862A-3.75 ASP 863A-3.77 PHE 864A-3.77
HER2	Lapatinib	−9.7	5	SER 728A-2.13 SER 728A-3.40 CYS 805A-3.63 THR 862A-2.81 ASP 863A-3.38	VAL 734A-3.93 LYS 753A-3.53 LEU 785A-3.88 LEU 796A-3.44 THR 798A-3.53 LEU 852A-3.77 THR 862A-3.90 ASP 863A-3.78
HER2	Neratinib	−9	5	SER 783A-3.24 ARG 849A-3.48 ARG 849A-2.17 ASN 850A-3.73 ASP 863A-2.22	VAL 734A-3.65 LYS 753A-3.40 LEU 796A-3.71 THR 798A-3.76 ARG 849A-3.91 LEU 852A-3.31

### Molecular dynamics simulations

In this study, we conducted molecular dynamics simulations (MDS) for 1,000 ns across three independent replicates for apo and holo states. This methodology ensures robust and reliable insights into the system’s behavior by accounting for potential variability in the trajectories. Each replicate represents a distinct simulation run, facilitating a comprehensive analysis of structural stability and conformational changes. Among the three 1,000 ns simulations, Run1 was identified as the most stable based on key stability parameters such as RMSD. Consequently, further detailed analyses were performed exclusively on Run1 to derive meaningful insights and ensure reliable conclusions from the study. The average values of RMSD, RMSF, SASA, and Rg for both the apo and holo forms are provided in Supplementary Table 4. Detailed RMSD graphs are presented in Supplementary Figures S6–S8.

### Molecular dynamics simulations (apo state)

**FIGURE 6:**
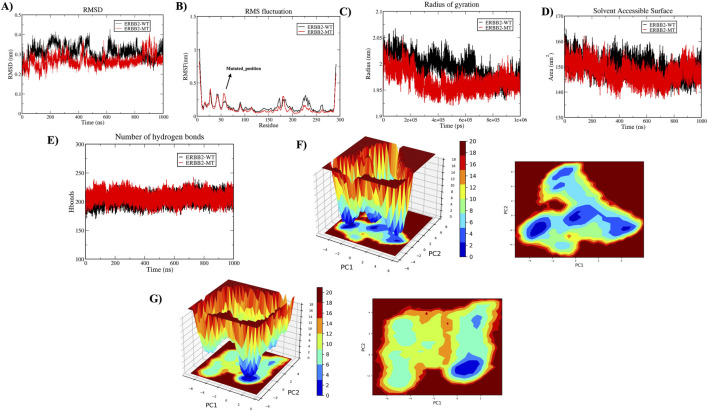
Molecular Dynamics Simulations in Apo State. The structural impact of wild-type (HER2-WT) and mutant-type (HER2-L755S) was analyzed over 1,000 ns. **(A)** Time plot of RMSD values for backbone atoms, with the X-axis indicating time in ns and the Y-axis indicating RMSD in nm. **(B)** RMSF values for Cα atoms over time, with residue number on the X-axis and RMSF (nm) on the Y-axis. **(C)** Rg plot, showing time in ps on the X-axis and Rg in nm on the Y-axis. **(D)** SASA plot, with the X-axis representing time in ns and the Y-axis indicating SASA in nm^2^. **(E)** Graph displaying intramolecular hydrogen bond interactions, with the Y-axis showing the number of hydrogen bonds and the X-axis representing time in ns. The wild type is represented in black, and the mutant type in red. **(F)** The Gibbs free energy landscape of 2D and 3D of HER2-WT were represented. **(G)** The Gibbs free energy landscape of 2D and 3D of HER2-MT were represented.

A 1000 ns trajectory of MDS Run 1 was analyzed to investigate the structural and functional changes induced by the HER2-L755S mutation compared to the wild-type (WT) using metrics such as RMSD, RMSF, Rg, intramolecular hydrogen bonds, and SASA. The RMSD of backbone atoms indicated overall stability, with the WT and HER2-L755S mutant proteins showing low RMSD values (≈0.35 nm and ≈ 0.3 nm, respectively). The WT protein exhibited moderate conformational changes within a range of less than 0.4 nm, suggesting that the mutation may cause local structural deviations without disrupting the global fold of the protein. Despite deviation during the simulation, both types remained stable. The RMSF of C-alpha atoms was used to examine flexibility changes in specific residues. Both WT and HER2-L755S mutants displayed RMSF values of ≈ 1 nm. Lower RMSF values in the mutant indicate reduced flexibility, suggesting a more rigid conformation. Slight changes in fluctuation between residues at positions 753–755 were observed for HER2-L755S, likely due to the introduction of a polar serine in place of hydrophobic leucine, causing local structural changes and stabilizing the mutant conformation. Rg, which measures protein compactness, showed values ranging from ≈1.95 to 2.1 nm, indicating a more compact structure. The HER2-L755S mutant exhibited more fluctuations, potentially leading to increased rigidity in specific regions and a more compact structure. SASA, which measures the protein’s exposed surface area, showed values of ≈ 160 nm^2^ for WT and ≈ 158 nm^2^ for HER2-L755S. A decrease in SASA for the mutant form compared to the WT indicates a more compact structure or a change that leads to buried hydrophobic residues, contributing to structural stability. The H-bond analysis, comparing the number of intramolecular hydrogen bonds between WT and HER2-L755S, showed a range of 175–225. The initial set of principal components, PC1 and PC2, were utilized to map the Gibbs free energy landscape, providing insights into the stability and energy dynamics of different states or transitions in the biomolecular system. The Gibbs free energy profile for each system was illustrated with color coding, representing Gibbs free energies in kJ/mol for each structural state, from lowest to highest. The energy values for HER2-WT (19.9 kJ/mol) and HER2-MT (21 kJ/mol) suggest that the mutant has a slightly higher binding energy (21 kJ/mol) compared to the WT (19.9 kJ/mol). This small difference could indicate a subtle decrease in stability in the mutant due to structural or conformational changes caused by the mutation. Detailed results of the apo state are mentioned in Figure 6.

### Molecular dynamics simulation analysis (holo state)

**FIGURE 7:**
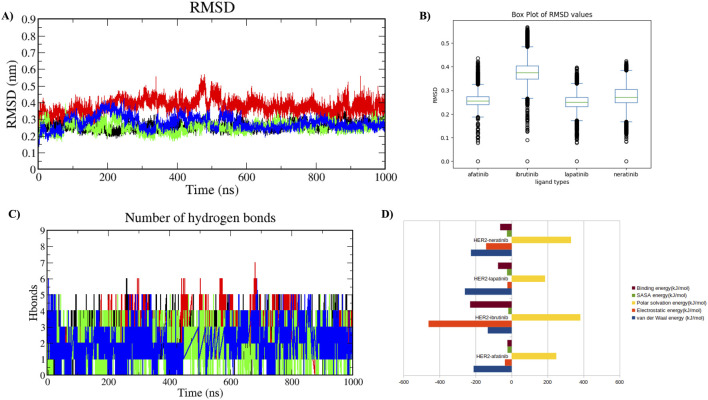
Molecular Dynamics Simulation in Holo State. Analysis of the protein-ligand complexes over 1,000 ns. Four different complexes were studied. **(A)** Time plot of RMSD values for backbone atoms, with the X-axis indicating time in ns and the Y-axis indicating RMSD in nm. **(B)** Boxplot displaying the respective RMSD values, with the X-axis denoting ligand types and the Y-axis denoting RMSD value. **(C)** Graph showing the total number of hydrogen bond interactions, with the Y-axis indicating the number of hydrogen bonds and the X-axis representing time in ns. HER2-L755S-afatinib is shown in black, HER2-L755S-ibrutinib in red, HER2-L755S-lapatinib in green, and HER2-L755S-neratinib in blue. **(D)** Bar plot displaying the overall binding energies for the four different complexes.

A comparative MDS analysis was performed on the HER2-L755S mutation with standard drugs (afatinib, neratinib, and lapatinib) and a virtual screening drug (ibrutinib) to evaluate the structural instability induced by the mutation in the HER2 protein. Four MDS runs, each lasting 1,000 ns, were conducted, assessing structural parameters such as RMSD, RMSF, Rg, SASA, and intermolecular hydrogen (H) bonds. Additional analyses included Principal Component Analysis (PCA) and Gibbs free energy landscape evaluation. RMSD values of the four HER2-drug complexes indicated the degree of structural deviation over time compared to their initial conformation. Lower RMSD values typically suggest a more stable complex, whereas higher values might indicate greater flexibility or structural changes. The RMSD values for the four complexes were as follows: HER2-L755S-afatinib (≈0.25 nm), HER2-L755S-ibrutinib (≈0.35 nm), HER2-L755S-lapatinib (≈0.25 nm), and HER2-L755S-neratinib (≈0.3 nm). All complexes exhibited low RMSD values, indicating maintained overall protein conformation. HER2-L755S-afatinib and HER2-L755S-lapatinib, with RMSD values of ≈0.25 nm, indicated stable binding and structural integrity. HER2-L755S-neratinib, with an RMSD of ≈0.3 nm, suggested moderate stability, while HER2-L755S-ibrutinib exhibited greater structural fluctuations with an RMSD of ≈0.35 nm (Figures 7A, B).

RMSF values for the four HER2-L755S-drug complexes reflected residue flexibility during the simulation. Higher RMSF values indicate regions of greater flexibility, while lower values suggest more rigid or stable regions. RMSF values were: HER2-L755S-afatinib (≈0.6 nm), HER2-L755S-ibrutinib (≈0.6 nm), HER2-L755S-lapatinib (≈0.5 nm), and HER2-L755S-neratinib (≈0.75 nm). HER2-L755S-lapatinib showed the lowest RMSF, indicating less flexibility. HER2-L755S-neratinib exhibited the highest RMSF, suggesting the greatest flexibility. The moderate RMSF for HER2-L755S-ibrutinib reflected its non-covalent binding mode, allowing some degree of movement. The detailed results of RMSF and boxplot were mentioned in the Supplementary Figures S9A, B. Rg values described overall size and compactness of the molecular complex. The Rg values were: HER2-L755S-afatinib (≈2.05 nm), HER2-L755S-lapatinib (≈2 nm), HER2-L755S-neratinib (≈2 nm), and HER2-L755S-ibrutinib (≈2.1 nm). HER2-L755S-lapatinib and HER2-L755S-neratinib showed the most compact conformations, while HER2-L755S-afatinib was slightly more open. HER2-L755S-ibrutinib exhibited the most extended conformation. SASA values measured the surface area accessible to solvent molecules. SASA values were: HER2-L755S-afatinib (≈165 nm^2^), HER2-L755S-lapatinib (≈160 nm^2^), HER2-L755S-neratinib (≈165 nm^2^), and HER2-L755S-ibrutinib (≈168 nm^2^). HER2-L755S-lapatinib was the most compact, with the least surface exposure. HER2-L755S-ibrutinib was the most exposed, indicating a less compact and more flexible complex. The Rg and SASA graphs were mentioned in Supplementary Figures S9C, D. Intermolecular H-bond analysis, essential for stabilizing protein-ligand interactions, revealed the following: HER2-L755S-afatinib (n = 5), HER2-L755S-ibrutinib (n = 7), HER2-L755S-lapatinib (n = 5), and HER2-L755S-neratinib (n = 5). The HER2-L755S-ibrutinib complex exhibited the highest number of hydrogen bonds. The MDS results for intermolecular H-bonds are detailed in Figure 7C. Hydrogen bond occupancy for the four complexes was assessed using Visual Molecular Dynamics (VMD), focusing on the last 500 ns of the MDS to capture stabilized interactions. Occupancy values indicated the proportion of time each bond met the defined criteria, providing insights into stabilized hydrogen bonding. This analysis revealed which hydrogen bonds were consistently maintained in the final phase, with occupancy values indicating the proportion of time each bond met the defined criteria. This selective analysis provided insights into stabilized hydrogen bonding, aids in understanding the structural integrity and interaction dynamics at the later simulation stages. In this study, HER2-afatinib found five H-bonds, highest occupancy is 72.69% (LIG994-side–ASP808-side), whereas HER2-ibrutinib found nine H-bonds, highest occupancy is 34.23% (LYS736-side–LIG994-side), HER2-lapatinib found seven H-bonds, highest occupancy is 54.30% (PHE731-Main–LIG994-side) and HER2-neratinib found 14 H-bonds, highest occupancy is 48.90% (LIG994-side–ASP845-side). A high occupancy suggests the H-bond is stable and persistent throughout the simulation. Detailed information on H-bond occupancy for the four complexes is provided in Supplementary Table 5. Binding free energy of the four complexes, estimated using MM-PBSA analysis, showed the following: HER2-L755S-afatinib (−23.48 ± 24.999 kJ/mol), HER2-L755S-lapatinib (−75.517 ± 24.188 kJ/mol), HER2-L755S-neratinib (−63.620 ± 49.573 kJ/mol), and HER2-L755S-ibrutinib (−230.422 ± 28.203 kJ/mol). HER2-L755S-ibrutinib demonstrated the strongest binding affinity. Detailed results are presented in Table 2 and Figure 7D.

**TABLE 2:** MM-PBSA analysis of HER2-L755S-Drug Complexes.

Complex name	Van der Waal energy (kJ/mol)	Electrostatic energy (kJ/mol)	Polar solvation energy (kJ/mol)	SASA energy (kJ/mol)	Binding energy (kJ/mol)
HER2-L755S-Afatinib	−211.071 ± 15.248	−37.411 ± 35.715	247.934 ± 5.204	−22.932 ± 1.191	−23.480 ± 4.999
HER2-L755S-Ibrutinib	−132.347 ± 27.274	−461.228 ± 30.447	381.712 ± 26.388	−18.559 ± 2.135	−230.422 ± 28.203
HER2-L755S-Lapatinib	−259.315 ± 10.985	−23.353 ± 22.992	185.664 ± 16.710	−25.219 ± 1.092	−75.517 ± 24.188
HER2-L755S-Neratinib	−225.834 ± 25.888	−141.806 ± 105.517	329.955 ± 86.932	−25.935 ± 2.614	−63.620 ± 49.573

**FIGURE 8:**
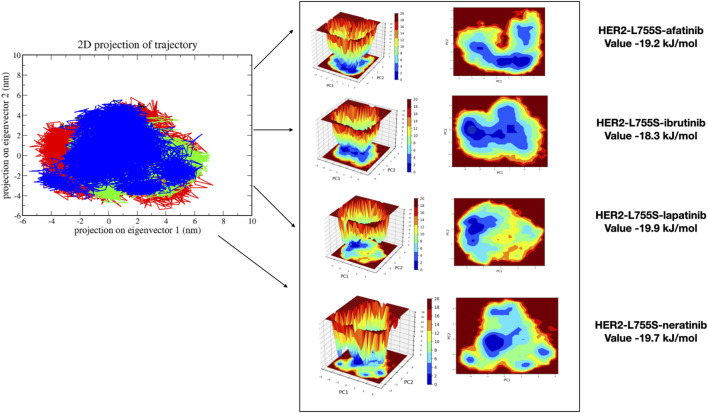
Principal Component Analysis and Gibbs free energy landscape of four complexes (HER2-L755S-afatinib, HER2-L755S-ibrutinib, HER2-L755S-lapatinib, and HER2-L755S-neratinib), exhibiting 2D projections of trajectories on the first two eigenvectors.

MDS analyzes the fundamental motions of biomolecular systems using Essential Dynamics (ED) or Principal Component Analysis (PCA). The MD trajectories of the four complexes are projected into the subspace defined by Principal Components (PCs) 1 and 2. ED analysis reveals that PC1 and PC2 capture the primary motions. All four complexes exhibited motion ranging from −5 to 7 on PC1 and -3 to 5 on PC2, occupying a larger conformational space and displaying varied conformations. Changes in cluster morphology were also observed in the conformational space for all complexes. The stability and energetics of different states or transitions within the biomolecular system were shown by projecting the Gibbs free energy landscape using the first pair of major components, PC1 and PC2. Color coding was used to depict each system’s Gibbs free energy landscape; the color bar shows the Gibbs free energies in kJ/mol for each structural state, going from lowest to highest. The energy values for the four complexes were as follows: HER2-L755S-afatinib (19.2 kJ/mol), HER2-L755S-ibrutinib (18.3 kJ/mol), HER2-L755S-lapatinib (19.9 kJ/mol), and HER2-L755S-neratinib (19.7 kJ/mol). Detailed results of 2D and 3D interactions are provided in Figure 8.

## Discussion

Globally, BC significantly impacts cancer incidence and mortality rates. Understanding the molecular mechanisms of BC is crucial for early detection, diagnosis, and treatment (Kashyap et al., 2022). This study analyzed BC mechanisms using computational methods, including screening for differentially expressed genes (DEGs), conducting MCODE analysis, identifying hub genes in the protein-protein interaction (PPI) network, validating them, and performing mutation analysis. These findings could aid in understanding BC development at the molecular level and identifying potential biomarkers for BC diagnosis and treatment. While numerous studies have focused on BC biomarkers (Zeng et al., 2021; Tian et al., 2020; Bankhead et al., 2017), none have specifically combined mutation analysis of hub genes with identifying alternative treatments tailored to these mutations for personalized medicine.

Treatment of BC, particularly in HER2-positive subtypes, has been revolutionized by targeted therapies like lapatinib, a dual tyrosine kinase inhibitor (TKI) that blocks HER2 and EGFR signaling (Yang et al., 2020). However, resistance to lapatinib poses a significant challenge, as many patients eventually relapse or fail to respond to the therapy. This study addresses these challenges by exploring gene expression profiling, hub gene identification, mutation analysis, and alternative therapeutic strategies to overcome lapatinib resistance. Gene expression profiling is a powerful tool that comprehensively examines transcriptional changes in cancer cells (Hijazo-Pechero et al., 2021).

We collected nine GEO datasets based on strict inclusion and exclusion criteria and performed a meta-analysis on these datasets (GSE3744, GSE26711, GSE5364, GSE16780, GSE15852, GSE17907, GSE42568, GSE36295, and GSE37751), identifying DEGs. This study identified several key genes in cancer development and progression by analyzing differential gene expression between BC samples and normal tissues. Meta-DEGs were compared to TCGA-BRCA and DisGeNET databases, identifying 61 overlapping genes. The functional analyses were performed on overlapping genes. Functional analyses were performed on these overlapping genes, revealing associations with kinase binding, signaling pathways, and cell-cycle functions, widely reported in BC studies (Song et al., 2014; Song and Farzaneh, 2021; Butti et al., 2018).

Network analysis identified MCODE clusters and hub genes. Hub genes with high connectivity within the molecular interaction network may be master regulators of oncogenic processes. The identified hub genes—*MYC*, *EGFR*, *CDKN2A*, *ERBB2*, *CDK1*, *E2F1*, *TOP2A*, *MDM2*, *TGFB1*, and *FOXM1*—play significant roles in BC progression and are potential biomarkers for diagnosis, prognosis, and therapy. These genes are involved in crucial pathways related to cell cycle regulation, proliferation, apoptosis, DNA repair, and metastasis, serving as biomarkers for prognosis and predicting breast cancer aggressiveness and potential outcomes (Duffy et al., 2021; Hsu and Hung, 2016; Milioli et al., 2020; Zhang et al., 2000; Almeida et al., 2014; Cheng et al., 2023). The top five hub genes were validated using TCGA expression data on different cancers and respective survival plots. Many of these genes are therapeutic targets, influencing the effectiveness of specific treatments like HER2-targeted therapies (*HER2*), CDK4/6 inhibitors (*CDKN2A*), and anthracyclines (*TOP2A*). From the COSMIC database, only HER2 among the top 20 mutations showed enrichment with a 5% mutation frequency. Additional analysis focused on the prevalent HER2 mutation at position L755S for further investigation.

Amplification or overexpression of HER2 occurs in approximately 15%–30% of BC cases and is associated with aggressive tumor behavior and poor prognosis. HER2-positive BC is often treated with targeted therapies, including trastuzumab (Herceptin) and lapatinib (Tykerb), a dual tyrosine kinase inhibitor of HER2 and EGFR (Dean, 2012). Lapatinib inhibits the intracellular tyrosine kinase domains of HER2 and EGFR, preventing phosphorylation and activation of downstream signaling pathways involved in cell proliferation and survival, such as the PI3K/AKT and MAPK pathways (Segovia-Mendoza et al., 2015). Despite its initial efficacy, resistance to lapatinib is a major clinical challenge.

MDS analysis of the apo state (WT and MT HER2-L755S) types was conducted over 1,000 ns, evaluating structural parameters such as RMSD, RMSF, Rg, SASA, and intramolecular H-bonds. The RMSD value was higher in MT HER2-L755S than WT, suggesting local instability with the rest of the protein compensating to maintain overall structural integrity (Yang et al., 2015). Lower RMSF in MT HER2-L755S compared to WT suggests reduced flexibility, potentially stabilizing the protein’s inactive form. Increased rigidity might stabilize a constitutively inactive conformation of HER2, promoting continuous signaling and cancer cell proliferation (Yang et al., 2015). The HER2-L755S mutation could decrease SASA by affecting hydrogen bonds and hydrophobic interactions, impacting protein folding and interaction with other molecules (Martínez, 2015). Hydrogen bonds are crucial for maintaining protein secondary and tertiary structures (Pace et al., 2014). Mutant HER2-L755S has increased potential for intramolecular hydrogen bonding due to serine, altering stability, flexibility, and conformational dynamics. The L755S mutation in the tyrosine kinase domain plays a crucial role in ATP binding and phosphorylation catalysis. Introducing polarity into a hydrophobic environment destabilizes local folding, potentially altering the ATP binding site and affecting kinase activity (Xu et al., 2017). In both HER-WT and HER2-MT, a reduction in hydrophobic interactions may impact the stability and conformation of the protein. These changes can disrupt the core structural integrity, potentially altering the drug binds to HER2 protein. These analysis were performed using ProteinTools (Ferruz et al., 2021). The HER2-WT has 38 contacts, and HER2-MT has 32 contacts. Hydrophobic contacts were reduced in the mutant type (Supplementary Table 6). The resistance of the HER2-L755S mutant to lapatinib is primarily due to structural and electrostatic changes at the binding site. Specifically, this mutation, which substitutes leucine for serine, alters the local conformation and dynamics around the binding pocket. These changes create hindrances that disrupt the optimal fit of lapatinib within the pocket. The HER2-L755S mutation may alter the local hydration environment around the binding pocket. Serine can bond with hydrogen and interact with water molecules differently than leucine, affecting local water networks. Such changes in hydration could alter binding energies and impact the stability of lapatinib’s binding. Each of these factors could combine to weaken the binding affinity of lapatinib for the HER2L755S, leading to the inhibitory effectiveness. These structural changes reduce lapatinib’s binding ability, making it less effective in inhibiting HER2 phosphorylation and downstream signaling, thereby promoting tumor cell proliferation and survival (Mariana et al., 2013). Cells with the L755S mutation exhibit reduced sensitivity to lapatinib but may remain sensitive to other inhibitors like neratinib and afatinib, which have different binding mechanisms and can inhibit the mutated HER2 receptor. To identify an alternative drug for HER2-L755S, 654 tyrosine kinase library compounds were screened. Among them, 344 passed the criteria for further processing, and ibrutinib was recognized as the top candidate based on binding affinity and MM-GBSA energies. Discovered in the early 2000s, ibrutinib irreversibly binds to BTK, blocking signals that promote B-cell cancer growth (Davids and Brown, 2014). Like afatinib and neratinib, ibrutinib is also an irreversible inhibitor (Dubovsky et al., 2013). This comparative study revealed atomistic insights into the binding mechanisms of sensitive drugs (neratinib and afatinib), resistance drugs (lapatinib), and virtual screening drugs (ibrutinib) upon HER2-L755S mutation. Molecular docking was performed on these four drugs, each exhibiting good binding affinity, and the best complexes were chosen based on hydrogen bond interactions. Ibrutinib showed the lowest binding energies among the three drugs. Further MDS analyses were conducted on these four HER2-L755S-drug complexes, examining various structural parameters such as RMSD, RMSF, Rg, SASA, intramolecular H-bonds, PCA, Gibbs free energy, and MM-PBSA analysis.

The RMSD measure assesses a molecular system’s stability and conformational changes. The HER2-L755S-afatinib and HER2-L755S-lapatinib complexes display stable RMSD values, implying strong binding and limited conformational fluctuation. HER2-L755S-neratinib and HER2-L755S-ibrutinib exhibit moderate stability, reflecting their strong but slightly more flexible interaction with HER2 (Carugo and Pongor, 2001). These RMSD values offer a snapshot of the relative stability and conformational dynamics of each HER2-L755S-drug complex. RMSF is another essential metric in MDS, analyzing the flexibility and movement of individual atoms or residues over time. HER2-L755S-lapatinib is the most stable complex, with minimal fluctuation in protein-ligand interactions. HER2-L755S-afatinib and HER2-L755S-ibrutinib exhibit moderate flexibility, indicating a balance between stability and movement. HER2-L755S-neratinib shows the highest flexibility, indicating dynamic regions within the complex despite covalent binding. These RMSF values provide insights into the stability and flexibility of different HER2-L755S-drug complexes, which could relate to their effectiveness or inhibition mechanisms (Martínez, 2015). Rg measures overall compactness and size in MDS (Lobanov et al., 2008). HER2-L755S-afatinib, HER2-L755S-lapatinib, and HER2-L755S-neratinib show compact conformations, reflecting strong interactions that maintain structural stability. HER2-L755S-ibrutinib shows a slightly more open conformation due to its binding nature. These Rg values complement RMSD and RMSF data, indicating that HER2-L755S-lapatinib and HER2-L755S-neratinib promote compact, stable conformations, while HER2-L755S-ibrutinib induces a more extended and flexible structure. SASA measures surface accessibility to solvent molecules (Durham et al., 2009). Low SASA values indicate regions buried or less accessible to solvent. HER2-L755S-lapatinib, HER2-L755S-afatinib, and HER2-L755S-neratinib form compact complexes with minimal solvent exposure, indicating tight and stable interactions. The HER2-L755S-ibrutinib complex is stable but retains some surface exposure, indicating flexibility despite strong binding. Hydrogen bonds are critical in stabilizing structure. HER2-L755S-ibrutinib forms the most hydrogen bonds, reflecting its covalent interaction strategy with HER2. Covalent inhibitors (afatinib and neratinib) rely on covalent bonds, while lapatinib depends on specificity and non-covalent bonding. These intermolecular H-bonds correlate well with overall complex stability (Pace et al., 2014). MM-PBSA analysis is essential for understanding biomolecular interaction energetics and protein-ligand interactions. The MM-PBSA value for the HER2-L755S-ibrutinib complex is highest compared to HER2-L755S-afatinib, HER2-L755S-neratinib, and HER2-L755S-lapatinib. In MDS, essential dynamics refers to determining and examining primary motion modes in a biomolecular system. All four complexes occupy larger space areas, suggesting more coordinated atomic movement during simulation. Gibbs free energy landscape analysis revealed lower energy minima for all complexes, indicating stable states (Maisuradze et al., 2009). In MDS, HER2-L755S-lapatinib stands out as the most favorable inhibitor structurally but shows reversible inhibition, leading to transient effects and resistance risks. HER2-L755S-afatinib and HER2-L755S-neratinib exhibit similar structural compactness and flexibility, ensuring long-term binding due to covalent inhibition. HER2-L755S-ibrutinib is also a covalent inhibitor to some degree of HER2, primarily targeting the BTK receptor. Ibrutinib binds to BTK’s cysteine-481 near the ATP binding domain, preventing phosphorylation and downstream pathway activation (Yang et al., 2013). This mechanism may apply to HER2 inhibition. Experimental studies suggest ibrutinib inhibits BC progression by converting myeloid-derived suppressor cells to dendritic cells (Varikuti et al., 2020). HER2-L755S poses significant treatment challenges due to lapatinib resistance. Understanding this mutation’s implications has led to alternative therapeutic strategies. Ibrutinib offers a promising avenue for overcoming resistance and improving outcomes for patients with the HER2-L755S mutation. Following a multi-step approach, further experimental validation is needed to assess ibrutinib’s efficacy against HER2 in BC.

## Limitations

Integrating computational findings into experimental research often presents challenges due to differences in scales, methodologies, and data availability. Computational methods offer insights into molecular interactions, dynamics, and energetics under controlled conditions. However, experimental results capture the complexity of biological systems, including environmental factors and allosteric effects. Bridging this gap requires identifying overlaps, refining computational models with experimental data, or designing complementary experiments to validate predictions in future studies. The above statement highlights the challenge on experimental methods, performed MDS in triplicate. This approach ensures reproducibility and strengthens the reliability of the results obtained from the simulations.

## Conclusion

Traditional drug discovery is a lengthy and costly endeavor, often requiring years and substantial financial investment. Computational techniques, such as *in silico* modeling, virtual screening, and statistical analysis, have significantly reduced these timelines by identifying potential drug candidates early in the process. In this study, integrating gene expression profiling, hub gene identification, and mutation analysis in BC provided valuable insights into the molecular mechanisms driving tumor progression and drug resistance. Network analysis revealed several hub genes crucial for regulating BC growth and metastasis, representing potential therapeutic targets and biomarkers for disease prognosis. Mutation analysis identified specific HER2-L755S genetic alterations associated with lapatinib resistance, contributing to therapeutic failure and underscoring the need for personalized approaches in treating BC. This study explores promising alternative therapies to overcome lapatinib resistance using molecular docking and dynamics studies to compare resistant and sensitive drugs. Ibrutinib demonstrated higher binding energies than control drugs (afatinib and neratinib) and lapatinib. In conclusion, this research underscores the importance of a multi-faceted approach, combining gene expression analysis, mutation identification, and targeted therapy development to address the challenges of drug resistance in BC.

## Data Availability

The datasets presented in this study can be found in online repositories. The names of the repository/repositories and accession number(s) can be found in the article/Supplementary Material.

## References

[B1] Abraham M. J. Murtola T. Schulz R. Páll S. Smith J. C. Hess B. (2015). GROMACS: high performance molecular simulations through multi-level parallelism from laptops to supercomputers. SoftwareX 1-2, 19–25. 10.1016/j.softx.2015.06.001

[B2] Aertgeerts K. Skene R. Yano J. Sang B.-C. Zou H. Snell G. (2011). Structural analysis of the mechanism of inhibition and allosteric activation of the kinase domain of HER2 protein. J. Biol. Chem. 286, 18756–18765. 10.1074/jbc.m110.206193 21454582 PMC3099692

[B3] Alam Md. S. Sultana A. Reza Md. S. Amanullah M. Kabir S. R. Mollah Md. N. H. (2022). Integrated bioinformatics and statistical approaches to explore molecular biomarkers for breast cancer diagnosis, prognosis and therapies. PLOS ONE 17, e0268967. 10.1371/journal.pone.0268967 35617355 PMC9135200

[B4] Alberts B. Johnson A. Lewis J. Raff M. Roberts K. Walter P. (2015). Studying gene expression and function. Nih. Gov. Available online at: https://www.ncbi.nlm.nih.gov/books/NBK26818/.

[B5] Almeida D. Gerhard R. Leitão D. Davilla C. Damasceno M. Schmitt F. (2014). Topoisomerase II-alfa gene as a predictive marker of response to anthracyclines in breast cancer. Pathology, Res. Pract. 210, 675–679. 10.1016/j.prp.2014.06.017 25042383

[B6] Amadei A. Linssen A. B. M. Berendsen H. J. C. (1993). Essential dynamics of proteins. Proteins Struct. Funct. Genet. 17, 412–425. 10.1002/prot.340170408 8108382

[B7] American Cancer Society (2023). Breast cancer statistics | how common is breast cancer? American Cancer Society. Available online at: https://www.cancer.org/cancer/types/breast-cancer/about/how-common-is-breast-cancer.html.

[B8] Arnold M. Morgan E. Rumgay H. Mafra A. Singh D. Laversanne M. (2022). Current and future burden of breast cancer: global statistics for 2020 and 2040. Breast 66, 15–23. 10.1016/j.breast.2022.08.010 36084384 PMC9465273

[B9] Bader G. D. Hogue C. W. (2003). An automated method for finding molecular complexes in large protein interaction networks. BMC Bioinforma. 4, 2. 10.1186/1471-2105-4-2 PMC149346 12525261

[B10] Bankhead P. Fernández J. A. McArt D. G. Boyle D. P. Li G. Loughrey M. B. (2017). Integrated tumor identification and automated scoring minimizes pathologist involvement and provides new insights to key biomarkers in breast cancer. Lab. Investig. 98, 15–26. 10.1038/labinvest.2017.131 29251737

[B11] Berendsen H. J. C. Postma J. P. M. van Gunsteren W. F. DiNola A. Haak J. R. (1984). Molecular dynamics with coupling to an external bath. J. Chem. Phys. 81, 3684–3690. 10.1063/1.448118

[B12] Bray F. Laversanne M. Sung H. Ferlay J. Siegel R. L. Soerjomataram I. (2024). Global cancer statistics 2022: GLOBOCAN estimates of incidence and mortality worldwide for 36 cancers in 185 countries. CA A cancer J. Clin. 74, 229–263. 10.3322/caac.21834 38572751

[B13] Breast cancer: MedlinePlus Genetics (2021). medlineplus.gov. Available online at: https://medlineplus.gov/genetics/condition/breast-cancer/.

[B14] Burley S. K. Berman H. M. Bhikadiya C. Bi C. Chen L. Di Costanzo L. (2018). RCSB Protein Data Bank: biological macromolecular structures enabling research and education in fundamental biology, biomedicine, biotechnology and energy. Nucleic Acids Res. 47, D464-D474–D474. 10.1093/nar/gky1004 PMC6324064 30357411

[B15] Butti R. Das S. Gunasekaran V. P. Yadav A. S. Kumar D. Kundu G. C. (2018). Receptor tyrosine kinases (RTKs) in breast cancer: signaling, therapeutic implications and challenges. Mol. Cancer 17, 34. 10.1186/s12943-018-0797-x 29455658 PMC5817867

[B16] Carugo O. Pongor S. (2001). A normalized root-mean-square distance for comparing protein three-dimensional structures. Protein Sci. A Publ. Protein Soc. 10, 1470–1473. 10.1110/ps.690101 PMC2374114 11420449

[B17] Cheng J. Ying L. Zhu L. Li X. Kołat D. Zhao L.-Y. (2023). Crucial role of the transcription factors family activator protein 2 in cancer: current clue and views. J. Transl. Med. 21, 371. 10.1186/s12967-023-04189-1 37291585 PMC10249218

[B18] Chin C.-H. Chen S.-H. Wu H.-H. Ho C.-W. Ko M.-T. Lin C.-Y. (2014). cytoHubba: identifying hub objects and sub-networks from complex interactome. BMC Syst. Biol. 8, S11. 10.1186/1752-0509-8-s4-s11 25521941 PMC4290687

[B19] Clough E. Barrett T. (2016). The gene expression omnibus database. Methods Mol. Biol. 1418, 93–110. 10.1007/978-1-4939-3578-9_5 27008011 PMC4944384

[B20] Daina A. Michielin O. Zoete V. (2017). SwissADME: a free web tool to evaluate pharmacokinetics, drug-likeness and medicinal Chemistry friendliness of small molecules. Sci. Rep. 7, 42717–42813. 10.1038/srep42717 28256516 PMC5335600

[B21] Davids M. S. Brown J. R. (2014). Ibrutinib: a first in class covalent inhibitor of Bruton's tyrosine kinase. Future Oncol. 10, 957–967. 10.2217/fon.14.51 24941982 PMC4632638

[B22] Dean L. (2012). Trastuzumab (herceptin) therapy and ERBB2 (HER2) genotype. Available online at: https://www.ncbi.nlm.nih.gov/books/NBK310376/.

[B23] Dubovsky J. A. Beckwith K. A. Natarajan G. Woyach J. A. Jaglowski S. Zhong Y. (2013). Ibrutinib is an irreversible molecular inhibitor of ITK driving a Th1-selective pressure in T lymphocytes. Blood 122, 2539–2549. 10.1182/blood-2013-06-507947 23886836 PMC3795457

[B24] Duffy M. J. O'Grady S. Tang M. Crown J. (2021). MYC as a target for cancer treatment. Cancer Treat. Rev. 94, 102154. 10.1016/j.ctrv.2021.102154 33524794

[B25] Durham E. Dorr B. Woetzel N. Staritzbichler R. Meiler J. (2009). Solvent accessible surface area approximations for rapid and accurate protein structure prediction. J. Mol. Model. 15, 1093–1108. 10.1007/s00894-009-0454-9 19234730 PMC2712621

[B26] ERBB2 - My Cancer Genome (2024). Available online at: https://www.mycancergenome.org/content/gene/erbb2/.

[B27] Essmann U. Perera L. Berkowitz M. L. Darden T. Lee H. Pedersen L. G. (1995). A smooth particle mesh Ewald method. J. Chem. Phys. 103, 8577–8593. 10.1063/1.470117

[B28] Feng Y. Spezia M. Huang S. Yuan C. Zeng Z. Zhang L. (2018). Breast cancer development and progression: risk factors, cancer stem cells, signaling pathways, genomics, and molecular pathogenesis. Genes and Dis. 5, 77–106. 10.1016/j.gendis.2018.05.001 PMC6147049 30258937

[B29] Ferruz N. Schmidt S. Höcker B. (2021). ProteinTools: a toolkit to analyze protein structures. Nucleic Acids Res. 49, W559–W566. 10.1093/nar/gkab375 34019657 PMC8262690

[B30] Forli S. Huey R. Pique M. E. Sanner M. F. Goodsell D. S. Olson A. J. (2016). Computational protein–ligand docking and virtual drug screening with the AutoDock suite. Nat. Protoc. 11, 905–919. 10.1038/nprot.2016.051 27077332 PMC4868550

[B31] Friesner R. A. Murphy R. B. Repasky M. P. Frye L. L. Greenwood J. R. Halgren T. A. (2006). Extra precision Glide: docking and scoring incorporating a model of hydrophobic enclosure for Protein−Ligand complexes. J. Med. Chem. 49, 6177–6196. 10.1021/jm051256o 17034125

[B32] Ge S. X. Jung D. Yao R. (2019). ShinyGO: a graphical gene-set enrichment tool for animals and plants. Bioinformatics 36, 2628–2629. 10.1093/bioinformatics/btz931 PMC7178415 31882993

[B33] Genheden S. Ryde U. (2015). The MM/PBSA and MM/GBSA methods to estimate ligand-binding affinities. Expert Opin. Drug Discov. 10, 449–461. 10.1517/17460441.2015.1032936 25835573 PMC4487606

[B34] Giaquinto A. N. Sung H. Newman L. A. Freedman R. A. Smith R. A. Star J. (2024). Breast cancer statistics 2024. CA A Cancer J. Clin. 74, 477–495. 10.3322/caac.21863 39352042

[B35] Helal M. Salauddin A. A. Mia M. F. U. Ema T. I. Yeasin R. B. Yeasin R. B. (2022). Oncoinformatic screening of the gene clusters involved in the HER2-positive breast cancer formation along with the *in silico* pharmacodynamic profiling of selective long-chain omega-3 fatty acids as the metastatic antagonists. Mol. Divers. 27, 2651–2672. 10.1007/s11030-022-10573-8 36445532

[B36] Hess B. Bekker H. Johannes F. Fraaije J. G. E. M. (1997). LINCS: a linear constraint solver for molecular simulations. J. Comput. Chem. 18, 1463–1472. 10.1002/(sici)1096-987x(199709)18:12<1463::aid-jcc4>3.0.co;2-h

[B37] Hijazo‐Pechero S. Alay A. Raúl H. M. Vilariño N. Muñoz-Pinedo C. Villanueva A. (2021). Gene expression profiling as a potential tool for precision oncology in non-small cell lung cancer. Cancers 13, 4734. 10.3390/cancers13194734 34638221 PMC8507534

[B38] Hsu J. L. Hung M.-C. (2016). The role of HER2, EGFR, and other receptor tyrosine kinases in breast cancer. Cancer Metastasis Rev. 35, 575–588. 10.1007/s10555-016-9649-6 27913999 PMC5215954

[B39] Humphrey W. Dalke A. Schulten K. (1996). VMD: Visual molecular dynamics. J. Mol. Graph. 14, 33–38. 10.1016/0263-7855(96)00018-5 8744570

[B40] Irizarry R. A. Bolstad B. M. Collin F. Cope L. M. Hobbs B. Speed T. P. (2003). Summaries of Affymetrix GeneChip probe level data. Nucleic Acids Res. 31, 15e15. 10.1093/nar/gng015 PMC150247 12582260

[B41] Ismail N. Z. Khairuddean M. Alidmat M. M. Abubakar S. Hasni A. (2024). Investigating the potential of mono-chalcone compounds in targeting breast cancer receptors through network pharmacology, molecular docking, molecular dynamics simulation, antiproliferative effects, and gene expressions. Biotech 14, 151. 10.1007/s13205-024-03991-y PMC11087420 38737798

[B42] Jiao Q. Bi L. Ren Y. Song S. Wang Q. Wang Y. (2018). Advances in studies of tyrosine kinase inhibitors and their acquired resistance. Mol. Cancer 17, 36. 10.1186/s12943-018-0801-5 29455664 PMC5817861

[B43] Kaplan W. Littlejohn T. G. (2001). Swiss-PDB viewer (deep view). Briefings Bioinforma. 2, 195–197. 10.1093/bib/2.2.195 11465736

[B44] Kashyap D. Pal D. Sharma R. Garg V. K. Goel N. Koundal D. (2022). Global increase in breast cancer incidence: risk factors and preventive measures. BioMed Res. Int. 2022, 9605439–9605516. 10.1155/2022/9605439 35480139 PMC9038417

[B45] Kim S. Thiessen P. A. Bolton E. E. Chen J. Fu G. Gindulyte A. (2015). PubChem substance and compound databases. Nucleic Acids Res. 44, D1202–D1213. 10.1093/nar/gkv951 26400175 PMC4702940

[B46] Kumari R. Kumar R. Lynn A. (2014). g_mmpbsa—a GROMACS tool for high-throughput MM-PBSA calculations. J. Chem. Inf. Model. 54, 1951–1962. 10.1021/ci500020m 24850022

[B47] Lánczky A. Győrffy B. (2021). Web-based survival analysis tool tailored for medical research (KMplot): development and implementation. J. Med. Internet Res. 23, e27633. 10.2196/27633 34309564 PMC8367126

[B48] Lee J. Franovic A. Shiotsu Y. Kim S. T. Kim K.-M. Banks K. C. (2019). Detection of ERBB2 (HER2) gene amplification events in cell-free DNA and response to anti-HER2 agents in a large asian cancer patient cohort. Front. Oncol. 9, 212. 10.3389/fonc.2019.00212 31019892 PMC6458313

[B49] Li J. Xiao Q. Bao Y. Wang W. Goh J. Wang P. (2019). HER2-L755S mutation induces hyperactive MAPK and PI3K-mTOR signaling, leading to resistance to HER2 tyrosine kinase inhibitor treatment. Cell Cycle 18, 1513–1522. 10.1080/15384101.2019.1624113 31135266 PMC6592242

[B50] Lipinski C. A. (2004). Lead- and drug-like compounds: the rule-of-five revolution. Drug Discov. Today Technol. 1, 337–341. 10.1016/j.ddtec.2004.11.007 24981612

[B51] Lobanov M.Yu. Bogatyreva N. S. Galzitskaya O. V. (2008). Radius of gyration as an indicator of protein structure compactness. Mol. Biol. 42, 623–628. 10.1134/S0026893308040195 18856071

[B52] Łukasiewicz S. Czeczelewski M. Forma A. Baj J. Sitarz R. Stanislawek A. (2021). Breast cancer—epidemiology, risk factors, classification, prognostic markers, and current treatment strategies—an updated review. Cancers 13, 4287. 10.3390/cancers13174287 34503097 PMC8428369

[B53] Maisuradze G. G. Liwo A. Scheraga H. A. (2009). Principal component analysis for protein folding dynamics. J. Mol. Biol. 385, 312–329. 10.1016/j.jmb.2008.10.018 18952103 PMC2652707

[B54] Mariana L. P. Verma C. S. Fuentes G. (2013). Differences in the binding affinities of ErbB family: heterogeneity in the prediction of resistance mutants. PLOS ONE 8, e77054. 10.1371/journal.pone.0077054 24194858 PMC3806757

[B55] Martínez L. (2015). Automatic identification of mobile and rigid substructures in molecular dynamics simulations and fractional structural fluctuation analysis. PLOS ONE 10, e0119264. 10.1371/journal.pone.0119264 25816325 PMC4376797

[B56] Mayo Clinic (2018). What your breast cancer type means. Rochester, Minnesota: Mayo Clinic. Available online at: https://www.mayoclinic.org/diseases-conditions/breast-cancer/in-depth/breast-cancer/art-20045654.

[B57] Mehrotra R. Yadav K. (2022). Breast cancer in India: present scenario and the challenges ahead. World J. Clin. Oncol. 13, 209–218. 10.5306/wjco.v13.i3.209 35433294 PMC8966510

[B58] Michael I. G. Rodin D. Pyatnitskiy M. A. Mackelprang M. Koman I. (2023). A review of HER2 overexpression and somatic mutations in cancers. Crit. Rev. Oncology/Hematology 186, 103997. 10.1016/j.critrevonc.2023.103997 37062337

[B59] Milioli H. H. Alexandrou S. Lim E. Caldon C. E. (2020). Cyclin E1 and cyclin E2 in ER+ breast cancer: prospects as biomarkers and therapeutic targets. Endocrine-Related Cancer 27, R93-R112–R112. 10.1530/erc-19-0501 32061162

[B61] Pace C. N. Fu H. Fryar K. L. Landua J. Trevino S. R. Schell D. (2014). Contribution of hydrogen bonds to protein stability. Protein Sci. A Publ. Protein Soc. 23, 652–661. 10.1002/pro.2449 PMC4005716 24591301

[B62] Pahuja K. B. Nguyen T. T. Jaiswal B. S. Prabhash K. Thaker T. M. Senger K. (2018). Actionable activating oncogenic ERBB2/HER2 transmembrane and juxtamembrane domain mutations. Cancer Cell 34, 792–806. 10.1016/j.ccell.2018.09.010 30449325 PMC6248889

[B63] Piñero J. Bravo À. Queralt-Rosinach N. Gutiérrez-Sacristán A. Deu-Pons J. Centeno E. (2016). DisGeNET: a comprehensive platform integrating information on human disease-associated genes and variants. Nucleic Acids Res. 45, D833-D839–D839. 10.1093/nar/gkw943 27924018 PMC5210640

[B64] Roos K. Wu C. Damm W. Reboul M. Stevenson J. M. Lu C. (2019). OPLS3e: Extending Force Field Coverage for Drug-Like Small Molecules. J. Chem. Theory Comput. 15, 1863–1874. 10.1021/acs.jctc.8b01026 30768902

[B65] Salentin S. Schreiber S. Haupt V. J. Adasme M. F. Schroeder M. (2015). PLIP: fully automated protein–ligand interaction profiler. Nucleic Acids Res. 43, W443–W447. 10.1093/nar/gkv315 25873628 PMC4489249

[B66] Schrödinger L. DeLano W. (2020). PyMOL. Available online at: http://www.pymol.org/pymol.

[B67] Segovia-Mendoza M. González-González M. E. Barrera D. Díaz L. García-Becerra R. (2015). Efficacy and mechanism of action of the tyrosine kinase inhibitors gefitinib, lapatinib and neratinib in the treatment of HER2-positive breast cancer: preclinical and clinical evidence. Am. J. cancer Res. 5, 2531–2561. 26609467 PMC4633889

[B68] Sharma P. Hu-Lieskovan S. Wargo J. A. Ribas A. (2017). Primary, adaptive, and acquired resistance to cancer immunotherapy. Cell 168, 707–723. 10.1016/j.cell.2017.01.017 28187290 PMC5391692

[B69] Shelley J. C. Cholleti A. Frye L. L. Greenwood J. R. Timlin M. R. Uchimaya M. (2007). Epik: a software program for pK(a) prediction and protonation state generation for drug-like molecules. J. Computer-Aided Mol. Des. 21, 681–691. 10.1007/s10822-007-9133-z 17899391

[B70] Song D. Cui S. Zhao G. Fan Z. Nolan K. Yang Y. (2014). Pathway-based analysis of breast cancer. Am. J. Transl. Res. 6, 302–311. 24936222 PMC4058311

[B71] Song K. Farzaneh M. (2021). Signaling pathways governing breast cancer stem cells behavior. Stem Cell Res. and Ther. 12, 245. 10.1186/s13287-021-02321-w 33863385 PMC8052733

[B72] Subramanian J. Katta A. Masood A. Vudem D. R. Kancha R. K. (2019). Emergence of ERBB2 mutation as a biomarker and an actionable target in solid cancers. Oncol. 24, e1303–e1314. 10.1634/theoncologist.2018-0845 PMC6975965 31292270

[B73] Sudhesh Dev S. Zainal Abidin S. A. Farghadani R. Othman I. Naidu R. (2021). Receptor tyrosine kinases and their signaling pathways as therapeutic targets of curcumin in cancer. Front. Pharmacol. 12, 772510. 10.3389/fphar.2021.772510 34867402 PMC8634471

[B74] Suvarna E. Setlur A. S. K C. M S. Niranjan V. (2024). Computational molecular perspectives on novel carbazole derivative as an anti-cancer molecule against CDK1 of breast and colorectal cancers via gene expression studies, novel two-way docking strategies, molecular mechanics and dynamics. Comput. Biol. Chem. 108, 107979. 10.1016/j.compbiolchem.2023.107979 37989072

[B75] Swain S. M. Shastry M. Hamilton E. (2022). Targeting HER2-positive breast cancer: advances and future directions. Nat. Rev. Drug Discov. 22, 101–126. 10.1038/s41573-022-00579-0 36344672 PMC9640784

[B76] Szklarczyk D. Kirsch R. Koutrouli M. Nastou K. Mehryary F. Hachilif R. (2022). The STRING database in 2023: protein–protein association networks and functional enrichment analyses for any sequenced genome of interest. Nucleic Acids Res. 51, D638–D646. 10.1093/nar/gkac1000 PMC9825434 36370105

[B77] Tan M. Yu D. (2013). Molecular mechanisms of ErbB2-mediated breast cancer chemoresistance. Landes: Bioscience. Available online at: https://www.ncbi.nlm.nih.gov/books/NBK6194/. 10.1007/978-0-387-74039-3_9 17993237

[B78] Tang Z. Li C. Kang B. Gao G. Li C. Zhang Z. (2017). GEPIA: a web server for cancer and normal gene expression profiling and interactive analyses. Nucleic Acids Res. 45, W98-W102–W102. 10.1093/nar/gkx247 28407145 PMC5570223

[B79] Tate J. G. Bamford S. Jubb H. C. Sondka Z. Beare D. M. Bindal N. (2018). COSMIC: the catalogue of somatic mutations in cancer. Nucleic Acids Res. 47, D941-D947–D947. 10.1093/nar/gky1015 PMC6323903 30371878

[B80] Tian Z. He W. Tang J. Liao X. Yang Q. Wu Y. (2020). Identification of important modules and biomarkers in breast cancer based on WGCNA. OncoTargets Ther. 13, 6805–6817. 10.2147/OTT.S258439 PMC7367932 32764968

[B81] Toro-Domínguez D. Martorell-Marugán J. López-Domínguez R. García-Moreno A. González-Rumayor V. Alarcón-Riquelme M. E. (2018). ImaGEO: integrative gene expression meta-analysis from GEO database. Bioinformatics 35, 880–882. 10.1093/bioinformatics/bty721 30137226

[B82] Uchida S. Kojima T. Sugino T. (2021). Clinicopathological features, tumor mutational burden, and tumour-infiltrating lymphocyte interplay in ERBB2-mutated breast cancer: *in silico* analysis. Pathology Oncol. Res. 27, 633243. 10.3389/pore.2021.633243 PMC8262144 34257600

[B83] Van Der Spoel D. Lindahl E. Hess B. Groenhof G. Mark A. E. Berendsen H. J. C. (2005). GROMACS: fast, flexible, and free. J. Comput. Chem. 26, 1701–1718. 10.1002/jcc.20291 16211538

[B84] Varikuti S. Singh B. Volpedo G. Ahirwar D. K. Jha B. K. Saljoughian N. (2020). Ibrutinib treatment inhibits breast cancer progression and metastasis by inducing conversion of myeloid-derived suppressor cells to dendritic cells. Br. J. Cancer 122, 1005–1013. 10.1038/s41416-020-0743-8 32025027 PMC7109110

[B85] Waarts M. R. Stonestrom A. J. Park Y. C. Levine R. L. (2022). Targeting mutations in cancer. J. Clin. Investigation 132, e154943. 10.1172/jci154943 PMC9012285 35426374

[B86] Waterhouse A. Bertoni M. Bienert S. Studer G. Tauriello G. Gumienny R. (2018). SWISS-MODEL: homology modelling of protein structures and complexes. Nucleic Acids Res. 46, W296-W303–W303. 10.1093/nar/gky427 29788355 PMC6030848

[B87] World Health Organisation (2023). Breast cancer. Available online at: https://www.who.int/news-room/fact-sheets/detail/breast-cancer#:∼:text=Overview.

[B88] Xu X. De Angelis C. Burke K. A. Nardone A. Hu H. Qin L. (2017). HER2 reactivation through acquisition of the HER2 L755S mutation as a mechanism of acquired resistance to HER2-targeted therapy in HER2+ breast cancer. Clin. Cancer Res. 23, 5123–5134. 10.1158/1078-0432.ccr-16-2191 28487443 PMC5762201

[B89] Yang B. Zhang H. Wang H. (2015). Atomistic insights into the lung cancer-associated L755P mutation in HER2 resistance to lapatinib: a molecular dynamics study. J. Mol. Model. 21, 24. 10.1007/s00894-015-2580-x 25620423

[B90] Yang L.-Q. Ji X.-L. Liu S.-Q. (2013). The free energy landscape of protein folding and dynamics: a global view. J. Biomol. Struct. Dyn. 31, 982–992. 10.1080/07391102.2012.748536 23297835

[B91] Yang X. Wu D. Yuan S. (2020). Tyrosine kinase inhibitors in the combination therapy of HER2 positive breast cancer. Technol. Cancer Res. and Treat. 19, 1533033820962140. 10.1177/1533033820962140 33034269 PMC7592330

[B92] Zeng X. Shi G. He Q. Zhu P. (2021). Screening and predicted value of potential biomarkers for breast cancer using bioinformatics analysis. Sci. Rep. 11, 20799. 10.1038/s41598-021-00268-9 34675265 PMC8531389

[B93] Zhang S. Y. Liu S. C. Al-Saleem L. F. Holloran D. Babb J. Guo X. (2000). E2F-1: a proliferative marker of breast neoplasia. Cancer Epidemiol. Biomarkers and Prev. 9, 395–401. Available online at: https://aacrjournals.org/cebp/article/9/4/395/109642/E2F-1-A-Proliferative-Marker-of-Breast-Neoplasia1. 10794484

[B94] Zoete V. Cuendet M. A. Grosdidier A. Michielin O. (2011). SwissParam: a fast force field generation tool for small organic molecules. J. Comput. Chem. 32, 2359–2368. 10.1002/jcc.21816 21541964

[B95] Zubair M. Wang S. Ali N. (2021). Advanced approaches to breast cancer classification and diagnosis. Front. Pharmacol. 11, 632079. 10.3389/fphar.2020.632079 33716731 PMC7952319

