# Supplementary material for: Computational molecular insights into ibrutinib as a potent inhibitor of HER2-L755S mutant in breast cancer: gene expression studies, virtual screening, docking, and molecular dynamics analysis
Source: Front Mol Biosci. 2025 Mar 19;12:1510896. doi: 10.3389/fmolb.2025.1510896 (PMC11962039; doi:10.3389/fmolb.2025.1510896)
Supplement: Supplementary file 1 [file DataSheet1.docx]

**Supplementary Information**

**Computational Molecular Insights into Ibrutinib as a Potent Inhibitor of HER2-L755S Mutant in Breast Cancer: Gene Expression Studies, Virtual Screening, Docking, and Molecular Dynamics Analysis**

**Supplementary Table 1.** Summary of gene expression datasets of Breast Cancer

| **Geo_accession** | **Experiment** | **Total_samples (available in the dataset)** | **Total_samples (Used in this study)** | **No of controls** | **No of cases** | **Tissue_type** | **Platform** | **Possible combination** | **Treatment_type (Yes/No)** |
| --- | --- | --- | --- | --- | --- | --- | --- | --- | --- |
| GSE3744 | Expression profiling by array | 47 | 47 | 7 | 40 | Breast_tissue | GPL570 [HG-U133_Plus_2] Affymetrix Human Genome U133 Plus 2.0 Array | Control: bulk normal breast or normal breast organoid; Case: breast tumor | No |
| GSE20711 | Expression profiling by array | 90 | 90 | 2 | 88 | Breast_tissue | GPL570 [HG-U133_Plus_2] Affymetrix Human Genome U133 Plus 2.0 Array | Control: Normal breast tissue; Case: Breast tumor | No |
| GSE5364 | Expression profiling by array | 341 | 196 | 13 | 183 | Breast_tissue | GPL96[HG-U133A] Affymetrix Human Genome U133A Array | Control: Breast Normal sample; Case: Breast tumor sample | No |
| GSE10780 | Expression profiling by array | 185 | 185 | 143 | 42 | Breast_tissue | GPL570 [HG-U133_Plus_2] Affymetrix Human Genome U133 Plus 2.0 Array | Control: Unremarkable breast ducts (Normal); Case: Invasive ductal carcinoma (IDC) | No |
| GSE15852 | Expression profiling by array | 86 | 86 | 43 | 43 | Breast_tissue | GPL96[HG-U133A] Affymetrix Human Genome U133A Array | Control: normal breast tissue; Case: breast tumor tissue | No |
| GSE17907 | Expression profiling by array | 109 | 55 | 4 | 51 | Breast_tissue | GPL570 [HG-U133_Plus_2] Affymetrix Human Genome U133 Plus 2.0 Array | Control: normal breast tissue; Case: breast tumor tissue | No |
| GSE42568 | Expression profiling by array | 121 | 121 | 17 | 104 | Breast_tissue | GPL570 [HG-U133_Plus_2] Affymetrix Human Genome U133 Plus 2.0 Array | Control: normal breast tissue; Case: breast tumor tissue | No |
| GSE36295 | Expression profiling by array | 50 | 50 | 5 | 45 | Breast_tissue | GPL6244[HuGene-1_0-st] Affymetrix Human Gene 1.0 ST Array [transcript (gene) version] | Control: normal breast tissue; Case: breast tumor tissue | No |
| GSE37751 | Expression profiling by array | 108 | 108 | 47 | 61 | Breast_tissue | GPL6244 [HuGene-1_0-st] Affymetrix Human Gene 1.0 ST Array [transcript (gene) version] | Control: normal breast tissue; Case: breast tumor tissue | No |

**Supplementary Table 2.** Virtual Screening and ADMET profiling of ibrutinib drug

| **Virtual Screening results** | |
| --- | --- |
| Mutation | L755S |
| Drug library | Tyrosine kinase library |
| XP G-score | -11.864 |
| Glide evdw | -55.231 |
| Glide ecoul | -6.934 |
| Glide energy | -62.255 |
| Glide emodel | -86.096 |
| MM-GBSA ΔG bind | -69.206 |
| **ADMET results** | |
| **Characteristics** | **Values** |
| Molecule | Ibrutinib |
| Canonical SMILES | C=CC(=O)N1CCC[C@H](C1)n1nc(c2c1ncnc2N)c1ccc(cc1)Oc1ccccc1 |
| Formula | C25H24N6O2 |
| MW | 440.5 |
| #Heavy atoms | 33 |
| #Aromatic heavy atoms | 21 |
| Fraction Csp3 | 0.2 |
| #Rotatable bonds | 6 |
| #H-bond acceptors | 5 |
| #H-bond donors | 1 |
| MR | 131.01 |
| TPSA | 99.16 |
| iLOGP | 3.8 |
| XLOGP3 | 3.57 |
| WLOGP | 3.84 |
| MLOGP | 2.84 |
| Silicos-IT Log P | 2.59 |
| Consensus Log P | 3.33 |
| ESOL Log S | -4.9 |
| ESOL Solubility (mg/ml) | 5.61E-03 |
| ESOL Solubility (mol/l) | 1.27E-05 |
| ESOL Class | Moderately soluble |
| Ali Log S | -5.34 |
| Ali Solubility (mg/ml) | 2.02E-03 |
| Ali Solubility (mol/l) | 4.59E-06 |
| Ali Class | Moderately soluble |
| Silicos-IT LogSw | -6.53 |
| Silicos-IT Solubility (mg/ml) | 1.30E-04 |
| Silicos-IT Solubility (mol/l) | 2.95E-07 |
| Silicos-IT class | Poorly soluble |
| GI absorption | High |
| BBB permeant | No |
| Pgp substrate | Yes |
| CYP1A2 inhibitor | No |
| CYP2C19 inhibitor | Yes |
| CYP2C9 inhibitor | Yes |
| CYP2D6 inhibitor | Yes |
| CYP3A4 inhibitor | Yes |
| log Kp (cm/s) | -6.45 |
| Lipinski #violations | 0 |
| Ghose #violations | 1 |
| Veber #violations | 0 |
| Egan #violations | 0 |
| Muegge #violations | 0 |
| Bioavailability Score | 0.55 |
| PAINS #alerts | 0 |
| Brenk #alerts | 1 |
| Leadlikeness #violations | 2 |
| Synthetic Accessibility | 3.94 |

**Supplementary Table 3.** Molecular docking results of triplicates in four HER2-L755S-ligand complexes.

| **Runs** | **Compound** | **Docking Score** | **Hydrogen Bonds** |
| --- | --- | --- | --- |
| **afatinib** | | | |
| 1 | afatinib | -8.4 | 3 |
| 2 | afatinib | -8.5 | 1 |
| 3 | afatinib* | -7.7 | 5 |
| **lapatinib** | | | |
| 1 | lapatinib | -10.1 | 3 |
| 2 | lapatinib | -8.9 | 3 |
| 3 | lapatinib* | -9.7 | 5 |
| **neratinib** | | | |
| 1 | neratinib | -7.7 | 4 |
| 2 | neratinib* | -9 | 5 |
| 3 | neratinib | -8.9 | 4 |
| **ibrutinib** | | | |
| 1 | ibrutinib* | -10.4 | 2 |
| 2 | ibrutinib | -10.3 | 1 |
| 3 | ibrutinib | -9 | 2 |

**^*indicates that these complex runs chosen for further analysis^**

**Supplementary Table 4.** Average values of RMSD, RMSF, Rg, SASA over 1000 ns of Apo and holo forms.

| **Name** | **RMSD (nm)** | | | **RMSF(nm)** | | | **Rg(nm)** | | | **SASA(nm)** | | |
| --- | --- | --- | --- | --- | --- | --- | --- | --- | --- | --- | --- | --- |
|  | **Run1** | **Run2** | **Run3** | **Run1** | **Run2** | **Run3** | **Run1** | **Run2** | **Run3** | **Run1** | **Run2** | **Run3** |
| Apo (HER2-WT) | 0.308 ± 0.03 | 0.284± 0.03 | 0.287± 0.03 | 0.151 ± 0.109 | 0.127 ± 0.103 | 0.133 ± 0.121 | 1.992 ± 0.02 | 1.984 ± 0.01 | 2.000 ± 0.01 | 150.020 ± 3.51 | 151.557± 3.01 | 149.000 ± 3.16 |
| Apo (HER2-MT) | 0.267 ± 0.02 | 0.284 ± 0.02 | 0.251 ± 0.02 | 0.121 ± 0.09 | 0.125 ± 0.09 | 0.138± 0.11 | 1.965 ± 0.01 | 1.982 ± 0.01 | 1.967 ± 0.01 | 147.72 ± 3.51 | 151.44 ± 2.73 | 149.08 ± 3.11 |
| Holo (HER2-afatinib) | 0.258 ± 0.02 | 0.245 ± 0.02 | 0.254 ± 0.05 | 0.114 ± 0.08 | 0.111 ± 0.06 | 0.126 ± 0.11 | 2.015 ± 0.01 | 2.008 ± 0.01 | 2.022 ± 0.01 | 154.31 ± 2.91 | 151.51 ± 2.83 | 151.94 ± 2.59 |
| Holo (HER2-ibrutinib) | 0.377 ± 0.04 | 0.324 ± 0.03 | 0.269 ± 0.06 | 0.139 ± 0.07 | 0.128 ± 0.11 | 0.160 ± 0.10 | 2.057 ± 0.01 | 2.014 ± 0.01 | 2.040 ± 0.03 | 155.053 ± 2.78 | 151.419 ± 2.78 | 153.33 ± 3.35 |
| Holo (HER2-lapatinib) | 0.252 ± 0.02 | 0.272 ± 0.04 | 0.171 ± 0.03 | 0.107 ± 0.09 | 0.116 ± 0.09 | 0.103 ± 0.10 | 2.005 ± 0.01 | 2.008 ± 0.01 | 2.021 ± 0.01 | 149.723 ±2.72 | 151.840 ± 2.650 | 152.47 ± 2.455 |
| Holo (HER2-neratinib) | 0.277 ± 0.03 | 0.277 ± 0.04 | 0.168 ± 0.02 | 0.121 ± 0.10 | 0.121 ± 0.10 | 0.104 ± 0.07 | 1.998 ± 0.01 | 2.020 ± 0.01 | 2.004 ± 0.01 | 152.012 ± 2.91 | 152.231 ± 2.63 | 153.353 ± 2.40 |

**Supplementary Table 5.** Hydrogen Bond occupancy analysis of four complexes

| **HER2-afatinib (Found 5 h-bonds)** | | |
| --- | --- | --- |
| **Donor** | **Acceptor** | **Occupancy** |
| LIG994-Side | ASP808-Side | 72.69% |
| CYS 805-Main | LIG994-Main | 22.67% |
| MET801-Main | LIG994-Main | 8.84% |
| LYS736-Side | LIG994-Main | 0.38% |
| LIG994-Side | LEU726-Main | 0.20% |
| **HER2-ibrutunib (Found 9 h-bonds)** | | |
| **Donor** | **Acceptor** | **Occupancy** |
| LYS736-Side | LIG994-Side | 34.23% |
| LYS736-Side | LIG994-Main | 31.59% |
| LIG994-Side | ASP863-Side | 1.04% |
| MET801-Main | LIG994-Side | 4.74% |
| ARG849-Side | LIG994-Side | 1.02% |
| LIG994-Side | THR862-Side | 0.54% |
| LIG994-Side | GLN799-Main | 0.72% |
| ASN850-Side | LIG994-Side | 4.78% |
| THR862-Side | LIG994Side | 0.02% |
| **HER2-lapatinib (Found 7 h-bonds)** | | |
| **Donor** | **Acceptor** | **Occupancy** |
| PHE731-Main | LIG994-Side | 54.30% |
| LIG994-Side | ARG849-Main | 9.36% |
| LIG994-Side | SER728-Side | 1.38% |
| GLY732-Main | LIG994-Side | 0.28% |
| SER728-Main | LIG994-Side | 0.04% |
| SER728-Side | LIG994-Side | 0.02% |
| LYS883-Side | LIG994-Side | 0.02% |
| **HER2-neratinib(Found 14 h-bonds)** | | |
| **Donor** | **Acceptor** | **Occupancy** |
| THR862-Side | LIG994-Side | 0.02% |
| LIG994-Side | LEU726-Main | 0.04% |
| LIG994-Side | VAL725-Main | 0.02% |
| SER728-Side | LIG994-Side | 0.10% |
| LIG994-Side | SER728-Side | 0.04% |
| MET801-Main | LIG994-Side | 0.02% |
| LIG994-Side | ARG849-Main | 20.63% |
| LIG994-Side | ASP845-Side | 48.90% |
| ARG849-Side | LIG994-Side | 1.40% |
| LIG994-Side | ASN850-Side | 15.13% |
| CYS805-Main | LIG994-Side | 0.02% |
| LYS753-Side | LIG994-Side | 0.18% |
| LYS736-Side | LIG994-Side | 0.06% |
| SER728-Side | LIG994-Main | 0.14% |

| **Cluster-ID** | **Area** | **Number of contacts** | **Contacts/Residue** | **Area/Residue (Å)** |
| --- | --- | --- | --- | --- |
| 1(HER2-WT) | 1714.08 | 38 | 3.8 | 45.11 |
| 1(HER2-MT) | 1358.81 | 32 | 3.56 | 42.46 |

**Supplementary Table 6.** Hydrophobic contact analysis of HER2-WT and HER2-MT.

**Supplementary Figures**

**Figure S1**. HER2 frequency mutation analysis using cosmic database. **A)** A top 20 most gene mutation frequency in breast cancer. **B)** The top most prevalent mutation in breast cancer.

**
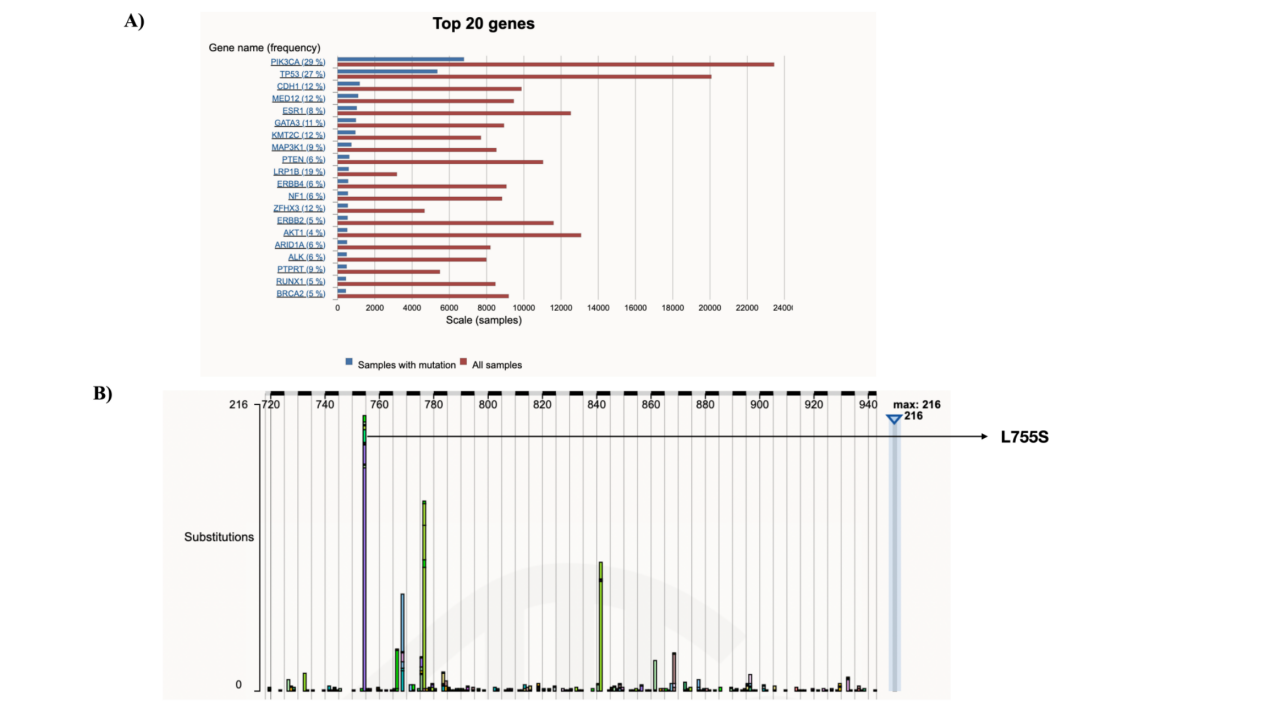
**

**Figure S2.The structure Assessment analysis**

1. The modeled structure was validated using Ramachandran Plot **B)** The modeled and original structures' aligned structures were visualized using PyMOL.

**
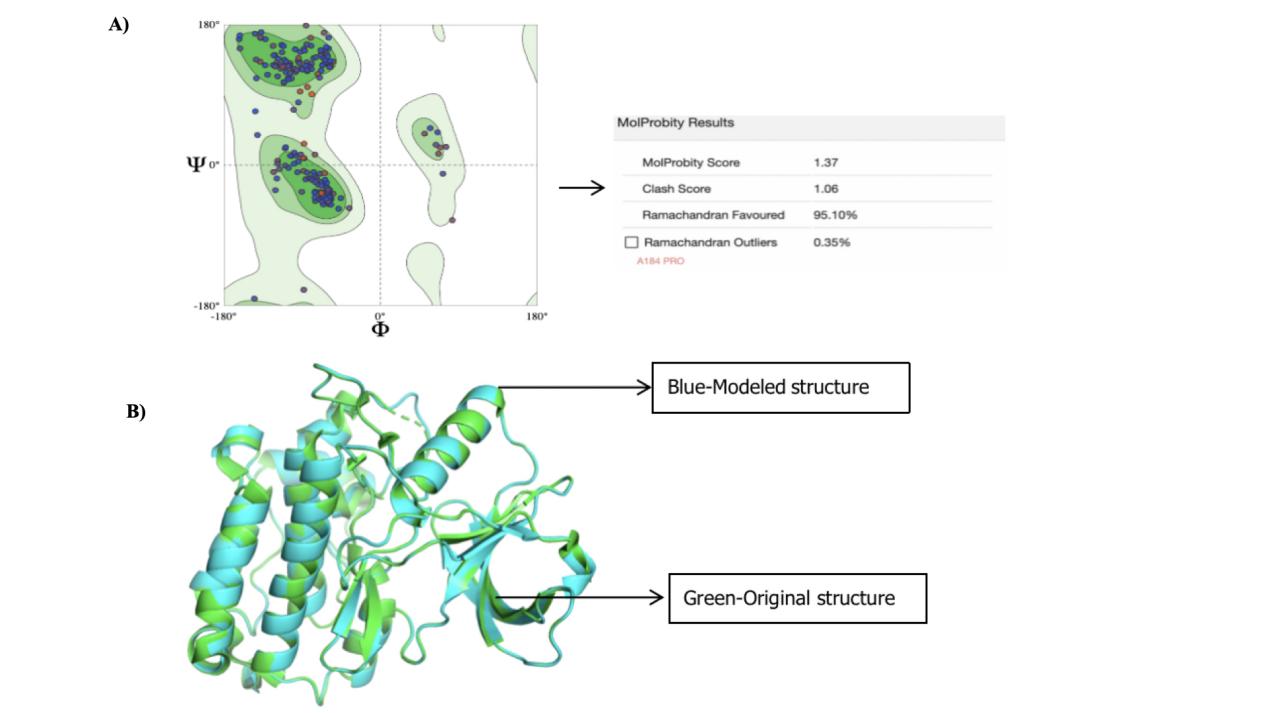
**

**Figure S3**. The boxplots of different expression datasets were analyzed. **A)** GSE3744 **B)** GSE20711 **C)** GSE5364 **D)** GSE10780 **E)** GSE15852 **F)** GSE17907


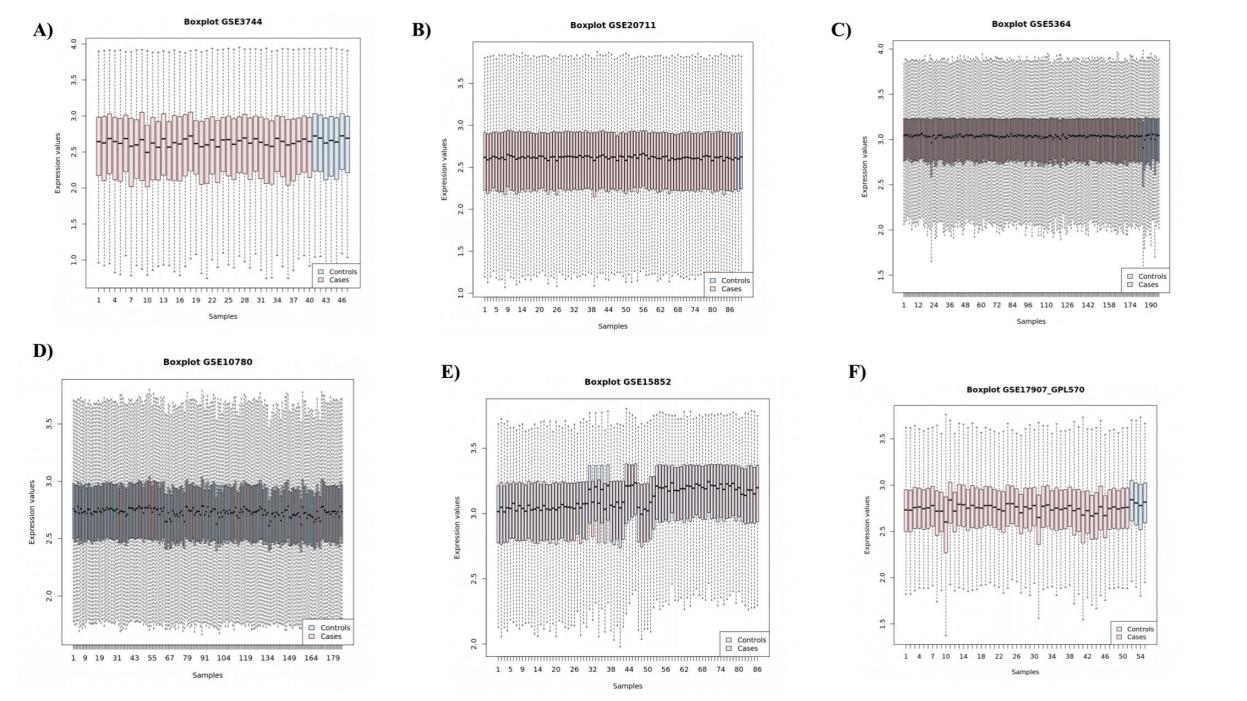


**Figure S4.** The boxplots different expression datasets and respective data quality were analyzed**. A)** GSE42568 **B)** GSE36295 **C)** GSE37751.


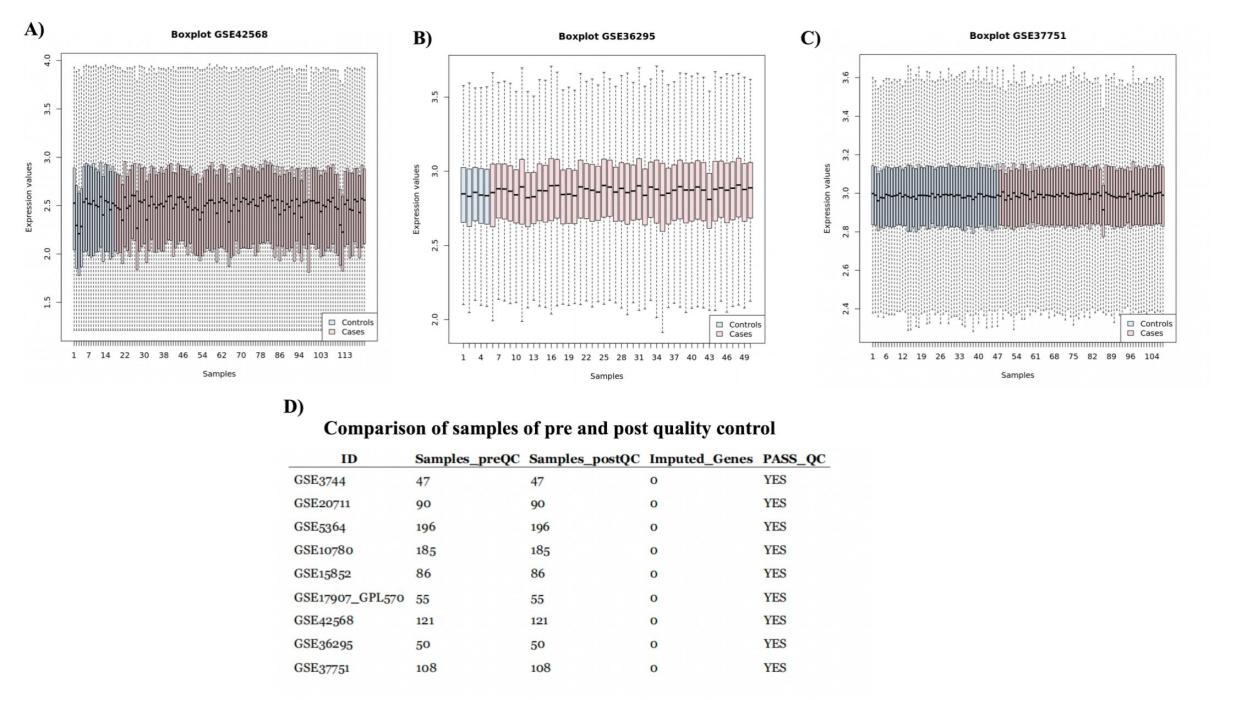


**Figure S5. Validation on top five hub genes.** The expression boxplot of all cancer expression and survival plot in five hub genes. **A)** *MYC* **B)** *EGFR* **C)** *CDKN2A* **D)** *ERBB2 (HER2)* **E)** *CDK1.*

*
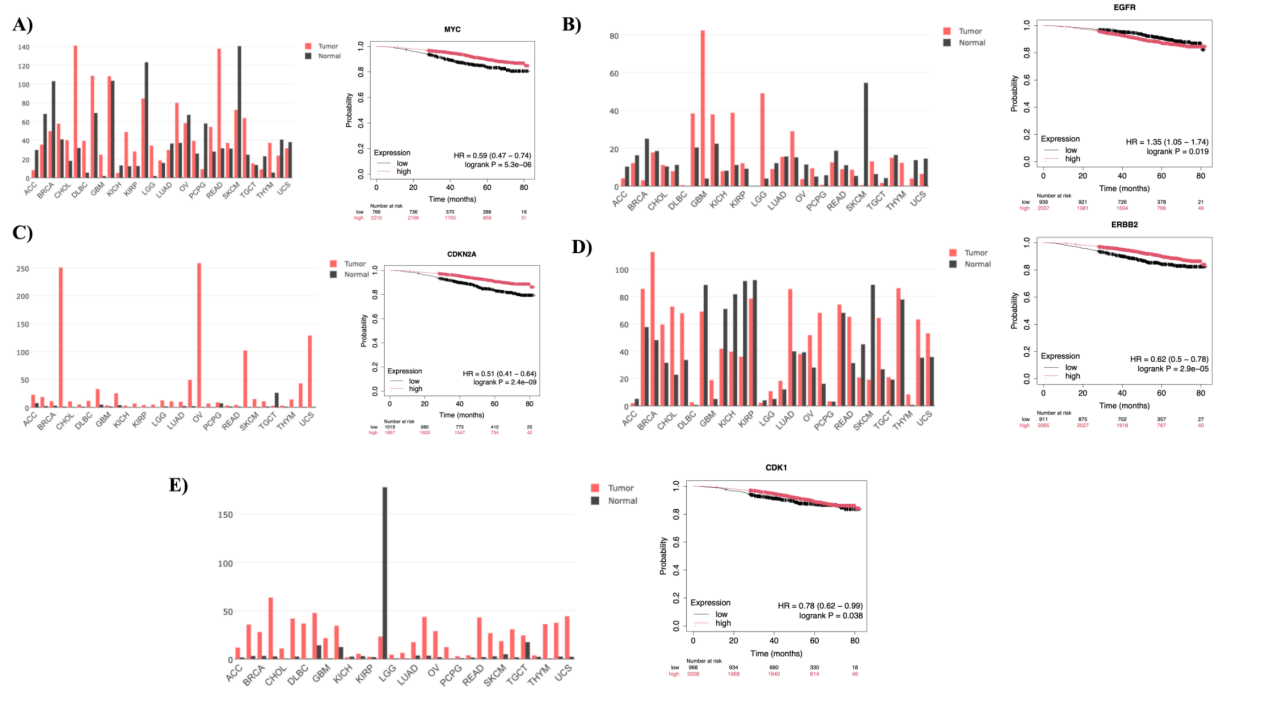
*

**Figure S6. The structural effects of wild-type (HER2-WT) and mutant-type (HER2-L755S) were examined over 1000 ns (triplicates).** Color Scheme is as follows: Run1-black, Run2-yellow, and Run3-brown. **A)** Time plot of RMSD values for HER2-WT backbone atoms, with the X-axis representing time in ns and the Y-axis representing RMSD in nm. **B)** Boxplot showing the RMSD values; the X-axis denotes model types, and the Y-axis denotes RMSD values. C) Time plot of RMSD values for HER2-MT backbone atoms, with the X-axis representing time in ns and the Y-axis representing RMSD in nm. D) Boxplot showing the RMSD values; the X-axis denotes model types, and the Y-axis denotes RMSD values


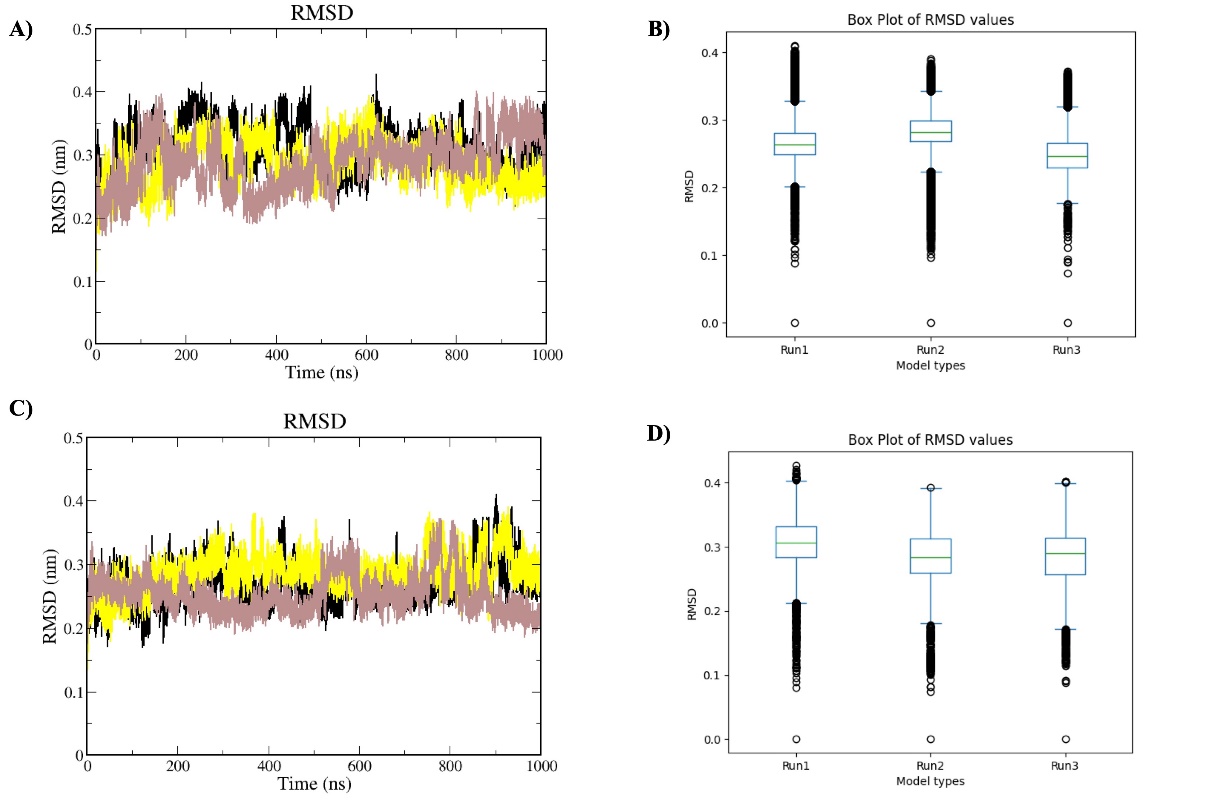


**Figure S7. The structural effects of various complexes were analyzed over 1000 ns (triplicates).** Color Scheme is as follows: Run1-black, Run2-yellow, and Run3-brown. A) Time plot of RMSD values for HER2-afatinib backbone atoms, with the X-axis representing time in ns and the Y-axis representing RMSD in nm. B) Boxplot showing the RMSD values; the X-axis denotes model types, and the Y-axis denotes RMSD values. C) Time plot of RMSD values for HER2-ibrutinib backbone atoms, with the X-axis representing time in ns and the Y-axis representing RMSD in nm. D) Boxplot showing the RMSD values; the X-axis denotes model types, and the Y-axis denotes RMSD values.


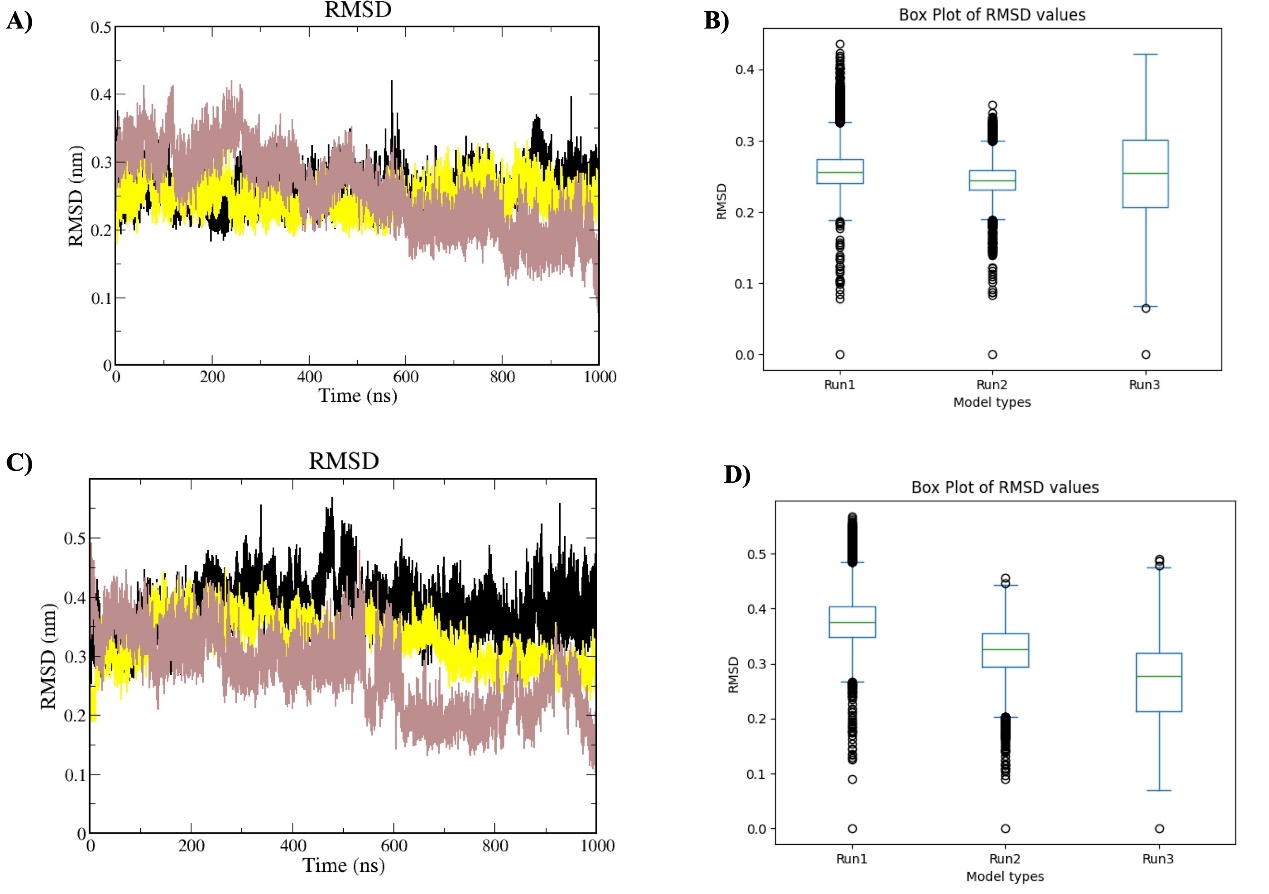


**Figure S8**. **The structural effects of various complexes were analyzed over 1000 ns (triplicates).** Color Scheme is as follows: Run1-black, Run2-yellow, and Run3-brown. **A)** Time plot of RMSD values for HER2-lapatinib backbone atoms, with the X-axis representing time in ns and the Y-axis representing RMSD in nm. **B)** Boxplot showing the RMSD values; the X-axis denotes model types, and the Y-axis denotes RMSD values. **C)** Time plot of RMSD values for HER2-neratinib backbone atoms, with the X-axis representing time in ns and the Y-axis representing RMSD in nm. **D)** Boxplot showing the RMSD values; the X-axis denotes model types, and the Y-axis denotes RMSD values.


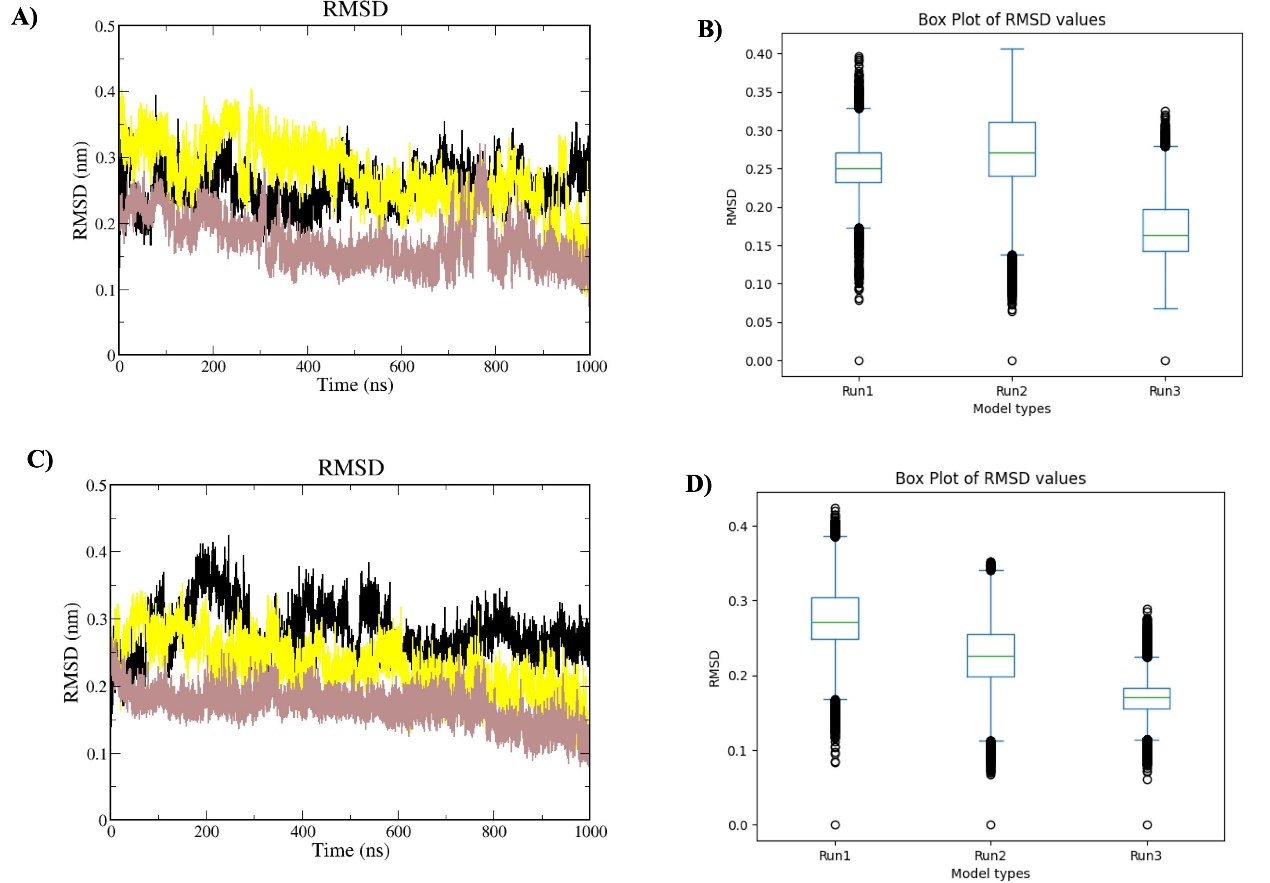


**Figure S9. Molecular Dynamics simulations (structural parameters) were used to analyze the protein-ligand complexes over 1000 ns.** Color Scheme is as follows: HER2-L755S-afatinib-black, HER2-L755S-ibrutinib-yellow, HER2-L755S-lapatinib-magenta, and HER2-L755S-neratinib-brown. A) RMSF plot, with the X-axis representing time in ns and the Y-axis representing RMSF in nm. B) Boxplot showing the RMSF values; the X-axis denotes ligand types, and the Y-axis denotes RMSF values. C) Rg plot, with the X-axis representing time in ps and the Y-axis representing Rg in nm. D) SASA plot, with the X-axis representing time in ns and the Y-axis representing SASA in nm².

**
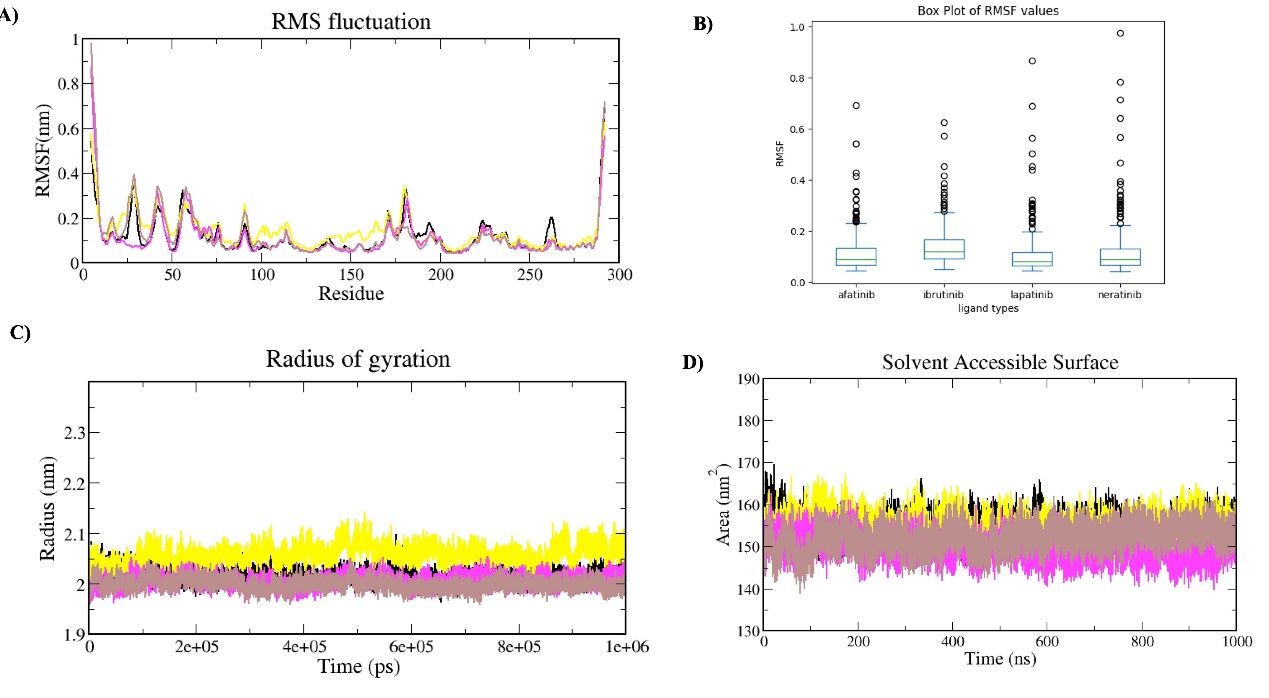
**
